# *Nocardia mangyaensis* NH1: A Biofertilizer Candidate with Tolerance to Pesticides, Heavy Metals and Antibiotics

**DOI:** 10.3390/microorganisms13122806

**Published:** 2025-12-09

**Authors:** Tatiana V. Shirshikova, Maria I. Markelova, Shanshan Zhou, Lydia M. Bogomolnaya, Margarita R. Sharipova, Irina V. Khilyas

**Affiliations:** 1 Institute of Fundamental Medicine and Biology, Kazan (Volga Region) Federal University, 420008 Kazan, Russia; 2 Laboratory of Multiomics Technologies of Living Systems, Institute Fundamental Medicine and Biology, Kazan (Volga Region) Federal University, 420008 Kazan, Russia; 3 State Key Laboratory of Microbial Diversity and Innovative Utilization, Institute of Microbiology, Chinese Academy of Sciences, Beijing 100101, China; 4 Department of Biomedical Sciences, Marshall University Joan C. Edwards School of Medicine, Huntington, WV 25755-0001, USA

**Keywords:** *Nocardia*, heavy metals, pesticides, antimicrobial resistance, biofertilizer

## Abstract

The extensive use of agrochemicals, heavy metals, and antibiotics in agriculture poses significant challenges to environmental sustainability and soil health. Plant growth-promoting bacteria (PGPB) offer a promising solution for sustainable agriculture; however, their selection requires careful evaluation of factors such as genome stability, metal tolerance, antibiotic resistance, and pesticide degradation capacity. This study characterizes the endolithic *Nocardia mangyaensis* NH1, focusing on its physiological and genomic features that enhance its potential as a biofertilizer in contaminated soils. Genomic analysis revealed a low number of antibiotic resistance genes with susceptibility to broad-spectrum antibiotics, minimizing the risk of horizontal gene transfer. The genome of *N. mangyaensis* NH1 contains two non-pathogenic genomic islands and prophage regions, with a CRISPR–Cas9 system. These findings highlight *N. mangyaensis* NH1 as a promising candidate for biofertilizers, combining pesticide and metal tolerance with genomic stability, thereby supporting sustainable agricultural practices and reducing environmental risks associated with agrochemical use.

## 1. Introduction

The extensive use of agrochemicals and pesticides, metal tolerance and antibiotic resistance represent significant challenges to modern agriculture and environmental sustainability [1]. The co-occurrence of heavy metals and pesticides in agricultural soils often leads to joint toxicity for plants and soil microorganisms, which can be either synergistic or antagonistic [2]. The agricultural application of metal-containing fertilizers and pesticides plays an important role in the maintenance and selection of antibiotic resistance among microorganisms, which in turn affects the formation of microbial communities and healthy plant rhizosphere [3].

One of the strategies employed in the practice of sustainable agriculture is the introduction of plant growth-promoting bacteria (PGPB), which serve to reduce reliance on chemical pesticides. However, the screening and selection of bacterial strains that are applicable for microbial-based fertilizers represent a significant challenge. Among bacteria, *Nocardia*, a genus of actinobacteria, are ubiquitous in a variety of ecological niches due to their high metabolic activity and capacity for adaptation to a range of stressors. A high resistance to heavy metals, the ability to biodegrade pesticides and plastics, synthesis of metabolites with activity against pathogenic bacteria and fungi, production of phytohormones and formation of nodules in actinorhizal plants were demonstrated for different strains of *Nocardia* [4,5]. Furthermore, the genomes of *Nocardia* species contain a variety of biosynthetic gene clusters (BGCs) responsible for the biosynthesis of bioactive secondary metabolites with antibacterial, antifungal and antiviral activities [6]. This metabolic potential, combined with their resilience to environmental stressors, positions *Nocardia* as a promising candidate for agrobiotechnological applications.

The tolerance of bacteria to heavy metals depends on the metal transportome, which represents a network of transport proteins involved in the uptake, efflux and sequestration of metal ions [7]. The control of metal homeostasis and cycling by transport systems enables microorganisms to survive in metal-rich environments. In addition to the significance of the diversity and the richness of the bacterial metal transportome, the antibiotic resistome is a pivotal factor in the distribution of antibiotic resistance genes within microbial communities in agricultural soils [7]. The goal in identification of novel PGPB candidates is to ensure that they will minimize environmental reservoirs of resistance genes, thereby preventing their transfer to phytopathogens and protecting agricultural ecosystems. The mechanisms of antibiotic and metal resistance co-selection have been extensively studied in bacteria from agricultural and industrial environments [8]. Furthermore, agricultural ecosystems are exposed to a variety of pesticides, the uncontrolled use of which has resulted in the bioaccumulation of these chemicals in plants, soil, air and water [2]. This has also led to an imbalance in microbial communities, with a predominance of phytopathogenic microorganisms.

Apart from above mentioned biosafety concerns related to pesticides, metal tolerance and antibiotic resistance, the genetic plasticity and stability of PGPB in the biofertilizers are also very important [9]. Bacterial genome integrity and stability are vital for their adaptation to a changing environment [10]. It is also crucial to maintain a balance between genome stability and plasticity when selecting bacterial strains for integration into agricultural practice. It is therefore essential to select PGPB as a component of biofertilizers based on investigation of their genome stability, metal transportome, antibiotic resistance and pesticide tolerance in order to advance sustainable agriculture. The objective of the present study was to characterize the physiological properties and genomic features of the endolithic *Nocardia mangyaensis* NH1 that contribute to its resistance to pesticides, heavy metals and antibiotics, thereby enhancing its potential as a biofertilizer in agricultural practice in poor and contaminated soils.

## 2. Materials and Methods

### 2.1. Bacterial Strain

Experiments were carried out using the endolithic strain *N. mangyaensis* NH1 isolated from the hydromagnesite mineral [11]. *N. mangyaensis* NH1 was grown on Mueller–Hinton agar. A bacterial suspension was then prepared in sterile 0.9% saline and left undisturbed to allow large clumps to settle. The resulting suspension was diluted to an OD600 of 0.1 or 0.5 according to MacFarland standards [12]. *N. mangyaensis* NH1 was deposited in the Russian National Collection of Industrial Microorganisms under collection number of VKPM: Ac-2226. The whole-genome shotgun project of *N. mangyaensis* NH1 was deposited at DDBJ/ENA/GenBank under accession JAUMIP000000000.

### 2.2. Assessment of Pesticide Resistance

*N. mangyaensis* NH1 was tested for sensitivity/resistance against field application rates of single fungicides (difenoconazole, fludioxonil, chlorothalonil), mixture of fungicides (tebuconazole and propiconazole) and herbicide (glufosinate ammonium) obtained from their respective manufacturers (SI_Table S1). Pesticide sterility was evaluated before their use in the experiments. Diluted pesticide solutions were freshly prepared prior to each experiment. The resulting suspension of *N. mangyaensis* NH1 was inoculated in Mueller–Hinton broth with the addition of pesticides at concentrations ranging from 1.0 to 4000 mg/L. Pesticide-free medium was used as a positive control. The incubation was performed at 30 °C for 72 h. The assessment of bacterial survival in the presence of pesticides was conducted through the enumeration of colony-forming units (CFU/mL). All experiments were performed in duplicate.

### 2.3. Heavy Metal Resistance

The minimum inhibitory concentration (MIC) to heavy metals was determined using a serial spot assay. The stock solutions of metals (NiSO_4_·7H_2_O, CoCl_2_·6H_2_O, K_2_Cr_2_O_7_, CuSO_4_·5H_2_O, ZnSO_4_, FeCl_3_·6H_2_O, AlCl_3_) were prepared in distilled water and filter-sterilized. The resulting suspensions and their appropriate serial dilutions were spotted onto agar plates supplemented with a gradient of metal concentrations (1.0–10.0 mM). Agar plates without metals served as a positive growth control. After incubation at 30 °C for 72 h a bacterial growth was assessed by enumerating CFU/mL. The MIC was defined as the lowest concentration of heavy metals that completely inhibited visible growth. All experiments were performed in triplicate.

The metal transportome of *N. mangyaensis* NH1 was predicted by using the TransportDB database 2.0 through the BLASTp v2.2.26 tool, with an E-value threshold of 1 × 10^−100^ [13]. The Antibacterial Biocide and Metal Resistance Genes Database (BacMet, experimentally confirmed database) v2.0 was used to search for resistance genes to biocides and metals through the BLASTp v2.2.26 tool, with an E-value threshold of 1 × 10^−50^ [14].

### 2.4. Antibiotic Susceptibility

The susceptibilities of *N. mangyaensis* NH1 were determined by E-test strip and disk diffusion assay on Mueller–Hinton agar (BD Difco) according to the Clinical and Laboratory Standards Institute (CLSI) guidelines (www.clsi.org). The resulting suspension of NH1 was streaked on the Mueller–Hinton agar using a sterile cotton-tipped applicator. E-test strips (Ezy MIC™, HIMEDIA, Kennett Square, PA, USA) and disks with antibiotics (MASTDISCS^®^ AST and Bio-Rad^®^ discs) were placed on the agar surface. The plates were incubated at 30 °C for 72 h, after which the inhibition zones surrounding the antimicrobial discs were measured. *N. mangyaensis* NH1 was categorized as sensitive or resistant according to previously published criteria [12,15,16]. Experiments were performed in triplicate. The Comprehensive Antibiotic Resistance Database (“CARD”) was used for predicting antibiotic resistance genes in the genome [17].

### 2.5. Genome Plasticity of N. mangyaensis NH1

The identification of signature genes for CRISPR–Cas types and subtypes was performed by using the online platform CRISPRCasFinder [18]. The genome of *N. mangyaensis* NH1 was analyzed using the PHASTEST (PHAge Search Tool with Enhanced Sequence Translation) web server to predict prophage sequences [19]. The classification of protein sequences into families was performed using the InterPro resource (https://www.ebi.ac.uk/interpro, accessed on 20 March 2025). The prediction of genomic islands in the *N. mangyaensis* NH1 genome was carried out with the IslandViewer webserver (http://www.pathogenomics.sfu.ca/islandviewer/, accessed on 20 March 2025) using the complete reference genome of *Nocardia asteroides* strain FDAARGOS 1485 [20].

### 2.6. Statistical Analysis of Data

The statistical analysis of the data was conducted using GraphPad Prism v. 8.0.1 (GraphPad Software, San Diego, CA, USA). The *p*-value was considered to be statistically significant if it was less than 0.05, and this was determined by the Kruskal–Wallis test.

## 3. Results

### 3.1. The Survival of N. mangyaensis NH1 in the Presence of Pesticides

**Figure 1:**
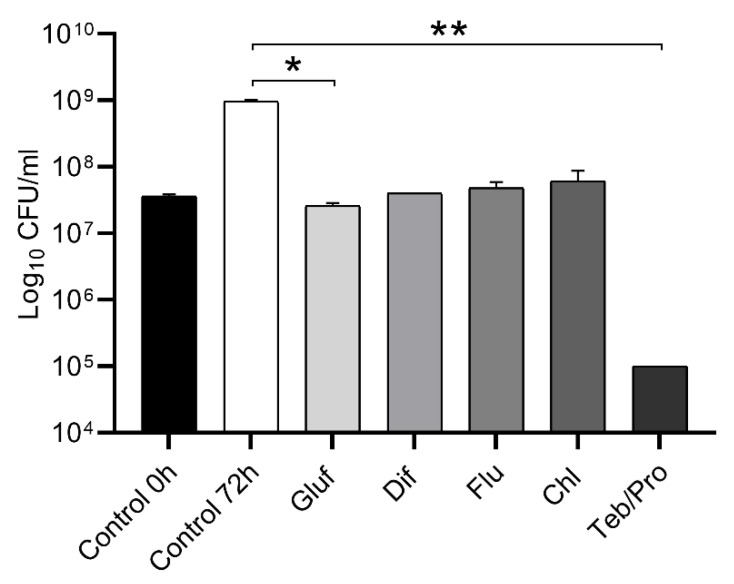
The survival of *N. mangyaensis* NH1 at field application rates of pesticides (Gluf, glufosinate-ammonium (2500 mg/L), Dif, difenoconazole (100 mg/L), Flu, fludioxonil (1500 mg/L), Chl, chlorothalonil (4000 mg/L) and Teb/Pro, tebuconazole and propiconazole (100/150 mg/L)) after 72 h growth at 30 °C in Mueller–Hinton medium. Values are means ± standard deviations. *—*p*-value < 0.05 and **—*p*-value < 0.01 are used to indicate significant differences between the control and experimental groups. All experiments were performed in duplicate.

The present study examined the viability of *N. mangyaensis* NH1 in pesticide solutions at field application rates over a 72 h period at 30 °C (Figure 1). The NH1 strain remained viable in a medium with glufosinate-ammonium, difenoconazole, fludioxonil and chlorothalonil with no significant reduction in cell count from the initial inoculation rate. However, statistically significant reduced bacterial growth of the NH1 strain was observed when exposed to glufosinate-ammonium in comparison with the untreated control. The fungicide mixture of tebuconazole and propiconazole (100 and 150 mg/L, respectively) was a notable exception, causing a statistically significant decrease in bacterial survival (Figure 1). The results revealed a positive correlation between the decrease in pesticide concentration and the increase in growth demonstrated by *N. mangyaensis* NH1 (SI_Figure S1).

### 3.2. Heavy Metal Resistance and Metal Transportome of N. mangyaensis NH1

*N. mangyaensis* NH1 demonstrated multiple resistance to heavy metals, with MIC values varying across a range of concentrations (Table 1, SI_Figure S2). The order of metal resistance, based on the MIC values, was as follows: Al^3+^ > Zn^2+^/Fe^3+^/Ni^2+^ > Co^2+^ > Cu^2+^/Cr_2_O_7_^2−^.

In order to determine the basic composition of the metal transportome involved in the metal resistance of *N. mangyaensis* NH1, the TransportDB 2.0 database was used, aiming to identify and assign putative function to all transport proteins. The following metal transporter groups were identified: ATP-dependent (ABC superfamily, P-type ATPase superfamily), ion channels (MIT, VIC), secondary transporters (ACR3, CaCA, CDF, CPA3, DASS, DMT, MFS, MOP, RND) and unclassified (MgtE) (Table 2).

Screening for heavy metal resistance genes in the genome of *N. mangyaensis* NH1 was conducted using the Antibacterial Biocide and Metal Resistance Genes (BacMet) database (Table 3). Seven genes encoding proteins that participate in cobalt, nickel, copper, iron, cadmium and lead resistance were identified, as well as one gene (*ideR*) that is involved in peroxide and naphthoquinone stress response. The results revealed that in the genome of *N. mangyaensis* NH1, two genes, *ctpD* and *kmtR*, are likely associated with resistance to cobalt (Co) and nickel (Ni). It was also suggested that three genes (*ideR*, *furA* and *acn*) might be involved in regulating of iron homeostasis (Table 3).

### 3.3. Antibiotic Resistance Profile of N. mangyaensis NH1

Genomic analysis using the Comprehensive Antibiotic Resistance Database (CARD) identified a limited resistance gene repertoire of *N. mangyaensis* NH1, which correlates with the observed phenotypic profile (Table 4). The antibiotic resistome of *N. mangyaensis* NH1 includes two genes, *vanY* and *vanW,* that encode proteins responsible for glycopeptide antibiotic resistance. Additionally the presence of one gene encoding rifampin monooxygenase, which participates in the inactivation of rifamycin, and the *aac(3)-I* gene, which encodes aminoglycoside 3-N-acetyltransferase and confers resistance to gentamicin and tobramycin were predicted. It is important to note that the results of the genomic analysis of *N. mangyaensis* NH1 are in good agreement with the phenotypic results of antibiotic susceptibility to glycopeptide antibiotics and aminoglycosides.

The present study also characterized the antibiotic resistance profile of *N. mangyaensis* NH1. The NH1 strain showed resistance to several antibiotics, including select aminoglycosides (kanamycin, tobramycin, streptomycin), cephalosporins (cefazolin), nitrofurans (nitrofurantoin), tetracyclines (tetracycline, doxycycline), and the glycopeptide vancomycin (SI_Table S2). Intermediate sensitivity was observed for gentamicin, erythromycin, and ciprofloxacin. The E-test determination of the minimum inhibitory concentration (MIC) confirmed susceptibility to these antibiotics at low concentrations (0.75, 1.5, and 1.0 µg/mL, respectively). The NH1 strain also demonstrated susceptibility to the aminoglycoside antibiotics neomycin and amikacin.

### 3.4. Genome Plasticity of N. mangyaensis NH1

**Figure 2:**
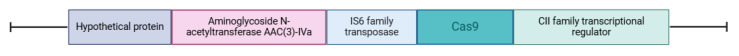
The genomic island in the genome of *N. mangyaensis* NH1 containing the type II CRISPR RNA-guided endonuclease Cas9.

To assess genome plasticity, a number of accessory genes beneficial under environmental conditions were explored. Five genomic islands (GIs) consisting of diverse insertion sequences (IS), integrases and transposases, were found in the genome of *N. mangyaensis* NH1 (SI_Table S3). The results showed the absence of pathogen-associated genes, curated virulence factors and resistance genes. One GI contains the Cas cluster. The Cas cluster, with an evidence level of 1 belonging to CAS-Type II-U, was detected in the contig NZ_JAUMIP010000046.1 (Figure 2). The contig contains five genes encoding Cas9 hypothetical protein, aminoglycoside N-acetyltransferase AAC(3)-IVa, IS6 family transposase, type II CRISPR RNA-guided endonuclease Cas9 and CII family transcriptional regulator. The Cas9 protein of *N. mangyaensis* NH1 was annotated by using InterPro, and the following domains were identified: HNH nuclease, CRISPR-associated endonuclease Cas9 with REC lobe and bridge helix, Cas9-type HNH domain and Ribonuclease H superfamily (Table 5). Additionally, our findings revealed that the genome of *N. mangyaensis* NH1 contains nine CRISPR arrays with direct repeats and spacers with an evidence level of 1, while one has an evidence level of 2 (SI_Table S4).

In this work, the composition of prophage genes and regions within the *N. mangyaensis* NH1 was studied using bioinformatic tools. The identification and annotation of prophage genes and regions within the *N. mangyaensis* NH1 genome revealed two regions containing incomplete and intact prophages, respectively, with total scores of 70 and 100 (SI_Figure S3, SI_Table S5). The average length of intact prophage sequences was found to be ∼12.5 kb. The GC content of the sequences analyzed was approximately 64% (SI_Table S5). Both prophage regions are located in the contig NZ_JAUMIP010000013.1 and are in close proximity to each other. The region 1 consists of nine proteins, including a regulated protein homologous to AraC family transcriptional regulator (PHAGE_Bordet_vB_BbrM_PHB04_NC_047861), a hypothetical protein, a phage-like protein homologous to gp132 (PHAGE_Mycoba_Cali_NC_011271), and six tail proteins homologous to minor tail proteins of different phages. The region 2 consists of 14 proteins, including two head proteins homologous to head-to-tail connector complex proteins (PHAGE_Strept_Rowa_NC_047906 and PHAGE_Rhodoc_Sleepyhead_NC_048782); four phage-like proteins homologous to pentapeptide repeat family proteins (PHAGE_Microc_MaMV_DC_NC_029002, PHAGE_Microc_MaMV_DC_NC_029002); a putative major virion structural protein (PHAGE_Brevib_Jenst_NC_028805) and a putative lysin (PHAGE_Gordon_GMA4_NC_030939); one lysis protein homologous to lysin B (PHAGE_Gordon_Attis_NC_041883); one repressor homologous to immunity repressor (PHAGE_Rhodoc_Jace_NC_047974); and four tail proteins homologous to minor tail proteins of different phages. In the *N. mangyaensis* NH1 genome, thirteen insertion elements (IS) belonging to eight IS families (IS5/IS1182, IS1380, IS630, IS3, ISAs1, IS6, IS21, IS30 and IS110 family transposases) were identified on the basis of the GenBank annotation (GCA_030519415.1). It was observed that, among all insertion sequences detected, those belonging to the IS5/IS1182 family transposase were found to be more prevalent.

## 4. Discussion

The agricultural sector is a major consumer of fertilizers, pesticides and soil supplements that contain heavy metals as impurities. The primary source of heavy metals (particularly Cd, Cu, Zn, Pb, Ni, As and Cr) are phosphorus fertilizers, which are extensively utilized in agricultural practices across numerous countries [21]. The application of pesticides, intended to treat plants against bacterial, fungal or insect diseases, is typically carried out through foliar spraying. However, this practice results in the wash-off of pesticides from treated plants and subsequent sorption of the pesticides onto soil particles [22]. The application of fertilizers and pesticides in greenhouse farming over an extended period has resulted in their accumulation, including heavy metals, and leading to a negative impact on soil fertility and crop productivity [23].

Over the last decade, there has been a global trend in agricultural practices towards the use of microbial inoculants, formulations or biofertilizers to enhance crop productivity and practice sustainable agriculture [24]. Plant growth-promoting bacteria (PGPB) are a core component of biofertilizers due to their beneficial properties such as secretion of extracellular lytic enzymes, production of phytohormones and increase in nutrient bioavailability [5]. Among PGPB, actinomycetes are beneficial microorganisms for agricultural application as biofertilizers due to their bioactive properties. The effectiveness of biofertilizer application depends on many factors, including biotic and abiotic factors present in the environment. Moreover, given the invasive nature of microbial inoculants toward indigenous microbial communities within agricultural systems, there is a growing concern regarding the assessment of unexpected consequences arising from their application [25]. Therefore, it is important to select microbial candidates for biofertilizers with consideration for their resistance to pesticides, heavy metals and antibiotics, as well as for their genome stability. This will increase their efficiency and minimize the distribution of resistant genes within the microbial communities in agricultural soils.

Members of the genus *Nocardia* are known for their biotechnological potential, multiple heavy metal resistance and ability to biodegrade organic compounds [26,27]. Recently, the genomic and metabolomic features of the endolithic strain *N. mangyaensis* NH1 were investigated, with the aim of determining their potential application beneficial in agricultural and environmental biotechnology [11]. A critical practical consideration is that microbial biofertilizers are typically applied as tank mixtures in combination with various pesticides. This study therefore presents an extensive analysis of the survival of *N. mangyaensis* NH1 at the field application rates of both single and mixture pesticides. It was established that *N. mangyaensis* NH1 maintained viability without a reduction in the initial CFU count throughout the extended incubation period with glufosinate-ammonium, difenoconazole, fludioxonil and chlorothalonil. This sustained survival during prolonged contact time with pesticides is critical when extending the time between tank mixing and field application is necessary.

The impact of fungicides on *Nocardia* species has been poorly explored in literature. In a recent study, it was demonstrated that one of the potential candidates for the bioremediation of environments contaminated with difenoconazole, which is categorized within the triazole group of chemicals, is strain *Pseudomonas putida* A-3, which possesses unique metabolic pathways for enzymatic biodegradation [28]. Several fungal strains, including *Fusarium oxysporum*, *Lentinula edodes*, *Penicillium brevicompactum* and *Lecanicillium sakseniae,* have been shown to biodegrade pesticides, including difenoconazole [29]. Biodegradation of the fungicide chlorothalonil, a tetrachlorinated benzonitrile, has been demonstrated for various bacterial genera (*Ochrobactrum*, *Shinella*, *Caulobacter*, *Rhizobium*, *Bordetella*, *Pseudoxanthomonas*, *Pseudomonas* and *Lysobacter*) [30]. Conversely, the effect of chlorothalonil on *Nocardia* strains remains to be elucidated. The biodegradation of fludioxonil, a fluorinated fungicide, was observed among numerous bacterial genera, including *Pseudomonas*, *Ochrobactrum* and *Comamonas* within agricultural soils [31].

Glufosinate-ammonium is an organophosphorus herbicide that was initially discovered as a tripeptide named bialaphos from actinomycetes belonging to the genus *Streptomyces* [32]. In order to control the growth of weeds that compete with the soybean plant, two bacterial genes (*hpat* and *mat*) encoding phosphinothricin and methionine sulfone acetyltransferases from bialaphos-resistant bacteria *Streptomyces* sp. strain AB3534 and *Nocardia* sp. strain AB2253 were selected to generate transgenic plants with confirmed resistance to glufosinate (PPT, phosphinothricin) [33,34]. Genome mining of *N. mangyaensis* NH1 revealed a sequence identity of 67% for GCN5-related N-acetyltransferases family (GNAT) with methionine sulfone acetyltransferase of *Nocardia* sp. strain AB2253. Although MTC of *N. mangyaensis* NH1 for glufosinate-ammonium remains below field application doses *in vitro*, the presence of the GNAT acetyltransferase (WP_302876268.1) implies potential for enzymatic detoxification of the herbicide under field conditions.

Agricultural management practice commonly involves simultaneous application of several pesticides. The present study evaluated the influence of a propiconazole and tebuconazole mixture on the viability of *N. mangyaensis* NH1. The results demonstrated a significant reduction in the initial CFU, indicating that separate application of the pesticides is necessary. For instance, the synergistic effect of a propiconazole and tebuconazole mixture in combination with the biocontrol strain *Bacillus subtilis* Czk1 was demonstrated against the fungus *Pyrrhoderma noxium*, which causes brown root rot of rubber trees [35]. The mechanism of the synergistic effect is thought to be the alteration of the metabolomic profile of the Czk1 strain, which leads to the synthesis of a diverse spectrum of metabolites, thereby increasing anti-phytopathogenic activity. Despite extensive use of tebuconazole and propiconazole in agricultural practice, their accumulation in soil has been demonstrated to induce shifts in microbial diversity and to promote the dissemination of a multidrug-resistant plasmid, as evidenced by the case of tebuconazole [36,37].

Along with pesticides, the issue of metal accumulation in agricultural ecosystems is a matter of concern. The heavy metal resistance profile of *N. mangyaensis* NH1 demonstrated distinct advantages over previously reported *Nocardia* strains. Although *Nocardia* MORSY2014 showed the effective removal of Ni^2+^, Cr^6+^ and Zn^2+^ ions [38] and Nigerian *Nocardia* isolates exhibited broad heavy metal tolerance [39], the NH1 strain shows a high tolerance to aluminium, zinc, nickel and ferric iron. In comparison with the pesticide-degrading strain of *N. harenae* JJB5 [40], which has been demonstrated to have a lower metal tolerance threshold, the NH1 strain exhibited balanced resilience to both heavy metals and pesticides.

An investigation of the metal transporter systems is crucial for the selection of PGPB for biofertilizers, since they directly impact the efficient regulation, uptake and detoxification of metals, as well as enhancing nutrient availability for plants. Genomic analyses indicate that *N. mangyaensis* NH1 encodes a large and diverse repertoire of potential metal transporters. A high number of transporters in the genome NH1 relate to the ABC superfamily. This family is known to participate in the uptake and efflux transport of a wide variety of molecules including nutrients, antibiotics, iron–siderophore complexes, virulence factors and envelope components [41,42]. Although transporters of the P-type ATPase superfamily are present in limited amount in the genome of *N. mangyaensis* NH1, they contain copper/mercury/heavy metal binding domains and catalyze the uptake and/or efflux of such cations as Mg^2+^, Cu^2+^, Ag^+^, Zn^2+^, Co^2+^, Pb^2+^, Ni^2+^ and Cd^2+^.

Among secondary transporters, the genome of *N. mangyaensis* NH1 is dominated by members of the MFS superfamily. The MFS transporters participate in the uptake and efflux of small molecules such as simple sugars, oligosaccharides, amino acids, Krebs cycle intermediates, antibiotics and nucleotides [43]. Some of the most represented members of the of metal transporters in the genome *N. mangyaensis* NH1 are proteins related to the RND superfamily, which are involved in the efflux of a range of substrates, including heavy metals, multiple drugs, antibiotics, lipooligosaccharides, lipids and pigments.

Three transport proteins relating to the Multidrug/Oligosaccharidyl-lipid/Polysaccharide (MOP) Superfamily were also identified in the genome of *N. mangyaensis* NH1 in limited amounts. They catalyze the efflux of primary and secondary metabolites such as terpenoids, phenols, flavonoids, nicotine, alkaloids, phytohormones, proanthocyanidin and anthocyanins. The MIT family (CorA/Mrs2) and VIC superfamily were both found in a single variant in the genome of *N. mangyaensis* NH1. These ion channels are identified as responsible for the import of Mg^2+^, Co^2+^, Ni^2+^, Zn^2+^ cations and are encoded by an ion-selective channel protein that facilitates the transport of K^+^, Na^+^ or Ca^2+^ [44].

The horizontal transfer of antibiotic resistance genes is known to be possible through co-occurrence with biocide/metal resistance genes found in bacterial chromosomes [45]. Consequently, the presence of metals in fertilizers or pesticide-contaminated soils could promote the spread of antibiotic resistance in the PGPB of biofertilizers and microbial communities of agricultural soils.

An investigation into the antibiotic susceptibility profile of potential PGPB biofertilizers is helpful for selecting bacteria lacking transferable antibiotic resistance genes, thereby reducing the risk of horizontal gene transfer. This is of particular relevance when considering the widespread use of livestock and poultry manure, which contributes to the dissemination of heavy metal and antibiotic resistance genes in agricultural soils [46]. The genomic analysis of *N. mangyaensis* NH1 revealed a limited repertoire of antibiotic resistance genes, identifying only two putative determinants: one conferring resistance to aminoglycosides and another to rifamycin. This finding provides a genotypic context for the observed phenotypic resistance profile of NH1 strain.

The antibiotic susceptibility profiles of numerous clinical isolates of the *Nocardia* species are well characterized [15,47,48,49,50]. However, the interpretation of the antibiotic susceptibility of *Nocardia* species remains a topic of debate, even according to widely accessible guidelines the European Committee on Antimicrobial Susceptibility Testing (EUCAST), unlike the actual restricted accessibility of the CLSI standards developed for clinical isolates [51]. Furthermore, the susceptibility criteria might vary significantly between clinical and environmental strains, as well as between different species and geographical origins, making direct comparisons challenging [15,52].

The phenotypic analysis showed that the NH1 strain is resistant to select aminoglycosides (kanamycin, tobramycin, streptomycin) but sensitive to neomycin and amikacin. This finding supports a recent study reporting that 91% of clinical *Nocardia* species are sensitive to amikacin [53]. It was confirmed that high-level aminoglycoside resistance in a clinical isolate of *N. farcinica* IFM 10580 is attributed to homozygous mutations in the 16S rRNA genes [54]. The resistance profile of NH1 showed a high degree of agreement with patterns observed in clinical isolates for other antibiotics. The resistance of *N. mangyaensis* NH1 to ceftazidime aligns with the resistance in 98% of the clinical isolates of *Nocardia* [55]. Similarly, *N. mangyaensis* NH1 resistance to nitrofurantoin and vancomycin is consistent with nocardial species-specific resistance reported in previous studies [56,57]. Furthermore, the high rates of intermediate resistance of *Nocardia* strains to the tetracycline class of antibiotic partially confirmed the resistance of *N. mangyaensis* NH1 to doxycycline [53].

The genomic analysis of *N. mangyaensis* NH1 revealed the presence of genomic islands. Nevertheless, no pathogen-associated genes, curated virulence factors or resistance genes were identified, which is an advantage for the application of *N. mangyaensis* NH1 in agriculture. Moreover, one genomic island in the genome of *N. mangyaensis* NH1 harbors genes encoding type II CRISPR (clustered regularly interspaced short palindromic repeats) RNA-guided endonuclease Cas9. It is well established that *cas*9 is the signature gene of type II CRISPR–Cas systems protecting bacterial genome against exogenous mobile genetic elements [58]. The absence of other genes such as *cas*1 and *cas*2 in the same locus or elsewhere in the genome suggests that the type II CRISPR–Cas system of *N. mangyaensis* NH1 is not complete. However, recent discoveries have revealed significant progress in the classification of CRISPR–Cas systems (now 7 types and 46 subtypes), with many rare variants, such as those in NH1, being identified from diverse environmental niches [59].

Insertion elements, which represent mobile DNA repeats, have been shown to be capable of copying and moving to different locations within the bacterial genome via a transposition mechanism. This capacity contributes to the plasticity of the bacterial genome and the regulation of genes [60]. As demonstrated in previous studies, there is significant variation in the abundance of insertion sequences (IS) among *Nocardia* genomes. This variation is indicative of divergent genomic capacities for acquiring exogenous DNA and tolerance to these mobile genetic elements [61]. The genome of *N. mangyaensis* NH1 was found to contain 13 ISs, whereas the genome of its closest species *N. mangyaensis* Y48 (GCA_001886715.1) contains 25 ISs belonging to six different families, with three common families (IS30, IS110, IS630). These differences in IS composition highlight the genomic diversity within closely related *Nocardia* strains and suggest varied capacities for genome plasticity and adaptation, which may influence their environmental fitness and functional potential.

Given the fact that prophages might induce genomic variability under stressful conditions or impose an influence on bacterial virulence, the analysis of prophage genes is a subject of growing interest. It is noteworthy that only two prophage regions, containing a total of 23 genes, were identified in *N. mangyaensis* NH1. This finding indicates that the *N. mangyaensis* NH1 genome faced limited horizontal gene transfer (HGT) over the course of evolution. For instance, a study of the genome of another strain of *Nocardia soli* Y48, isolated from soils contaminated with crude oil in the Qinghai-Tibetan Plateau, predicted 17 GIs consisting of 173 genes, suggesting frequent HGT events [62]. Moreover, the genome of *N. mangyaensis* NH1 could be characterized as a relatively stable due to the presence of a limited number of prophages lacking phage attachment sites (*attL*/*attR*) and integrases, as well as a potentially active CRISPR–Cas system. However, further studies are necessary to fully elucidate the dynamics of these genetic elements in this species.

## 5. Conclusions

This study provides a comprehensive analysis of the physiological and genetic characteristics of the endolithic bacterium *N. mangyaensis* NH1 as a promising biofertilizer candidate. The NH1 strain demonstrates remarkable multi-stress resistance to pesticides and heavy metals, supported by a diverse metal transportome. The genomic safety of *N. mangyaensis* NH1 was confirmed by a minimal set of chromosomal antibiotic resistance genes and stable genomic islands lacking pathogenicity factors. The compatibility of *N. mangyaensis* NH1 with key pesticides, including glufosinate-ammonium, difenoconazole, chlorothalonil and fludioxonil, further supports its practical application.

## Figures and Tables

**Table 1:** The minimum inhibitory concentration (MIC) of heavy metals for *N. mangyaensis* NH1.

Resistance Level	Metal Ions	MIC (mM)
Highest	Al^3+^	10
High	Zn^2+^, Fe^3+^, Ni^2+^	7
Medium	Co^2+^	5
Lowest	Cu^2+^, Cr_2_O_7_^2−^	4

**Table 2:** The metal transportome of *Nocardia mangyaensis* NH1.

Metal Transporter Type/Family	Number of Transporters
**I ATP-Dependent**	**116**
P-type ATPase (P-ATPase) Superfamily	5
ATP-binding cassette (ABC) Superfamily	111
**II Ion Channels**	**2**
CorA/Mrs2 Metal Ion Transporter (MIT) Family	1
Voltage-gated Ion Channel (VIC) Superfamily	1
**III Secondary Transporters**	**50**
Arsenical Resistance-3 (ACR3) Family	1
Ca^2+^: Cation Antiporter (CaCA) Family	1
Cation Diffusion Facilitator (CDF) Family	1
Monovalent Cation (K^+^ or Na^+^): Proton Antiporter-3 (CPA3) Family	2
Divalent Anion:Na^+^ Symporter (DASS) Family	1
Drug/Metabolite Transporter (DMT) Superfamily	1
Major Facilitator Superfamily (MFS)	26
Multidrug/Oligosaccharidyl-lipid/Polysaccharide (MOP) Flippase Superfamily	3
Resistance-Nodulation-Cell Division (RND) Superfamily	14
**IV Unclassified**	**2**
Mg^2+^ Transporter-E (MgtE) Family	2
**Total**	**170**

**Table 3:** Prediction of antibacterial biocide and metal resistance genes in the genome of *Nocardia mangyaensis* NH1.

Gene	ID	Species	Metal	Query	% Identity	N Identity	Length	E Value
*ctpD*	A0R3A7	*Mycobacterium smegmatis*	Cobalt (Co), Nickel (Ni)	WP_302872979.1	63.1	395	626	0
*mctB*	P64883	*Mycobacterium tuberculosis*	Copper (Cu)	WP_302872657.1	46.8	146	312	3.67 × 10^−80^
*furA*	P0A582	*Mycobacterium tuberculosis*	Iron (Fe)	WP_071931421.1	72.1	101	140	6.78 × 10^−72^
*ideR*	P0A672	*Mycobacterium tuberculosis*	Iron (Fe), Hydrogen Peroxide (H_2_O_2_) [class: Peroxides], Plumbagin [class: Naphthoquinone]	WP_071929125.1	73.9	170	230	1.65 × 10^−199^
*nmtR*	O69711	*Mycobacterium tuberculosis*	Nickel (Ni), Cadmium (Cd), Lead (Pb)	WP_302872707.1	75.63	90	119	1.99 × 10^−63^
*kmtR*	O53838	*Mycobacterium tuberculosis*	Nickel (Ni), Cobalt (Co)	WP_302871740.1	77.876	88	113	3.60 × 10^−59^
*acn*	O53166	*Mycobacterium tuberculosis* H37Rv	Iron (Fe)	WP_302870970.1	79.38	743	936	0

**Table 4:** The CARD-based antibiotic resistome prediction of *Nocardia mangyaensis* NH1.

RGI Critera	ARO Term	Detection Criteria	AMR Gene Family	Drug Class	Resistance Mechanism	% Identity of Matching Region	% Length of Reference Sequence	Predicted phenotype (ResFinder)
Strict	*vanY* gene in *vanB* cluster	protein homolog model	*vanY*, glycopeptide resistance gene cluster	glycopeptide antibiotic	antibiotic target alteration	32.17	64.18	-
Strict	*vanY*	protein homolog model	*vanW*, glycopeptide resistance gene cluster	glycopeptide antibiotic	antibiotic target alteration	28.91	166.22	-
Strict	*Nocardia farcinica rox*	protein homolog model	rifampin monooxygenase	rifamycin antibiotic	antibiotic inactivation	71.61	100.21	-
Strict	*aac(3)-IVa*	protein homolog model	AAC(3)	aminoglycoside antibiotic	antibiotic inactivation	98.84	103.49	Gentamicin, Tobramycin

Antibiotic Resistance Ontology (ARO), Resistance Gene Identifier (RGI). *aac(3)-IVa* is a plasmid-encoded aminoglycoside acetyltransferase in *E. coli*, *C. jejuni* and *P. stutzeri. Nocardia farcinica rox* is a rifampin monooxygenase that inactivates rifampin.

**Table 5:** InterPro annotation for the type II CRISPR RNA-guided endonuclease Cas9 (WP_302876581.1) of *N. mangyaensis* NH1.

Accession	Short Name	Accession
IPR003615	HNH_nuc	HNH nuclease
IPR028629	Cas9	CRISPR-associated endonuclease Cas9
IPR033114	HNH_CAS9	Cas9-type HNH domain
IPR032240	Cas9_REC	CRISPR-associated endonuclease Cas9, REC lobe
IPR032239	Cas9-BH	CRISPR-associated endonuclease Cas9, bridge helix
IPR036397	RNaseH_sf	Ribonuclease H superfamily

## Data Availability

The original contributions presented in this study are included in the article/Supplementary Materials. Further inquiries can be directed to the corresponding author.

## Supplementary Materials

The following supporting information can be downloaded at: https://www.mdpi.com/article/10.3390/microorganisms13122806/s1, Table S1: Agrochemicals used in the present study; Figure S1: The pesticide resistance of *N. mangyaensis* NH1; Figure S2: The heavy metal resistance of *N. mangyaensis* NH1; Table S2: *Nocardia mangyaensis* NH1 antibiotic susceptibility; Table S3: Predicted genomic islands and virulence/resistance genes in the genome of *N. mangyaensis* NH1. The prediction was performed by integrating three different methods (IslandPath-DIMOB, SIGI-HMM, and IslandPick) using the IslandViewer webserver; Table S4: Summary of information on CRISPR arrays and cas gene clusters in the genome of *N. mangyaensis* NH1; Figure S3: Genome map (A) and linear genome sequence (B) of *N. mangyaensis* NH1; predicted phage genes and regions positioned relative to each other across the entire genome; Table S5: Prediction of prophage sequences in the genome of *N. mangyaensis* NH1 was performed by using the PHASTEST web server.
