# Supplementary material for: Nocardia mangyaensis NH1: A Biofertilizer Candidate with Tolerance to Pesticides, Heavy Metals and Antibiotics
Source: Microorganisms. 2025 Dec 9;13(12):2806. doi: 10.3390/microorganisms13122806 (PMC12735748; doi:10.3390/microorganisms13122806)
Supplement: Supplementary file 1 [file microorganisms-13-02806-s001.zip › microorganisms-3958514-supplementary.pdf]

Supplementary Information

Nocardia mangyaensis NH1: a biofertilizer candidate with tolerance to pesticides, heavy metals and antibiotics

Tatiana V. Shirshikova[1], Maria I. Markelova[2], Shanshan Zhou[3], Lydia M. Bogomolnaya[4*], Margarita R. Sharipova[1], Irina V. Khilyas[1*]

[1] Institute of Fundamental Medicine and Biology, Kazan (Volga Region) Federal University, Kazan, Russian Federation
[2] Laboratory of Multiomics Technologies of Living Systems, Institute Fundamental Medicine and Biology, Kazan (Volga Region) Federal University, Kazan, Russian Federation
[3] State Key Laboratory of Microbial Diversity and Innovative Utilization, Institute of Microbiology, Chinese Academy of Sciences, Beijing, China
[4] Department of Biomedical Sciences, Marshall University Joan C Edwards School of Medicine, Huntington, USA

* Correspondence: irina.khilyas@gmail.com

Supporting Figure S1-S3
Supporting Table S1-S5

Table S1. Agrochemicals used in the present study.

Characteristics	Fungicides				Herbicides
	Single compounds			Mixture of compounds	Single compounds
Common name	Difenoconazole	Fludioxonil	Chlorothalonil	Tebuconazole and Propiconazole	Glufosinate-ammonium
Chemical name	1-[[2-[2-chloro-4-(4-chlorophenoxy)phenyl]- 4-methyl-1,3-dioxolan- 2-yl]methyl]-1,2,4-tria- zole	4-(2,2-difluoro-1,3-benzodioxol- 4-yl)-1H-pyr- role-3-car-bonitrile	2,4,5,6-tetrachloro-benzene-1,3-dicar-bonitrile	1-(4-chlorophenyl)-4,4-dimethyl-3- (1,2,4-triazol-1-ylmethyl)pentan-3- ol / 1-[[2-(2,4-dichlorophenyl)-4- propyl-1,3-dioxolan-2-yl]methyl]- 1,2,4-triazole	azanium;2-amino-4-[hydroxy(methyl)phos-phoryl]butanoate
Chemical class	Dioxolanes	Benzodioxoles	Dinitrile	Tertiary alcohol / cyclic ketal	Organophosphorus
Chemical for-mula	$C_{19}H_{17}Cl_2N_3O_3$	$C_{12}H_6F_2N_2O_2$	$C_8Cl_4N_2$	$C_{16}H_{22}ClN_3O$ / $C_{15}H_{17}Cl_2N_3O_2$	$C_5H_{15}N_2O_4P$
Molecular weight (g/mol)	406.3	248.18	265.9	307.82 / 342.2	198.16
Solubility	Water	Water	Water, acetone, methanol, etc.	Water / Water, ethanol, acetone, toluene, and octanol	Water
Recommended dose (g/L)	0.1	1.5	4	0.1/0.15	2.5
Source	Raek, KE ®, Avgust Crop Protec- tion, Russia	Sincler ®, CK, Avgust Crop Protec-tion, Russia	Talant ®, CK, Avgust Crop Pro- tection, Russia	Kolosal ®, Pro, KME, Protection, Russia	Basta ®, BP Basf

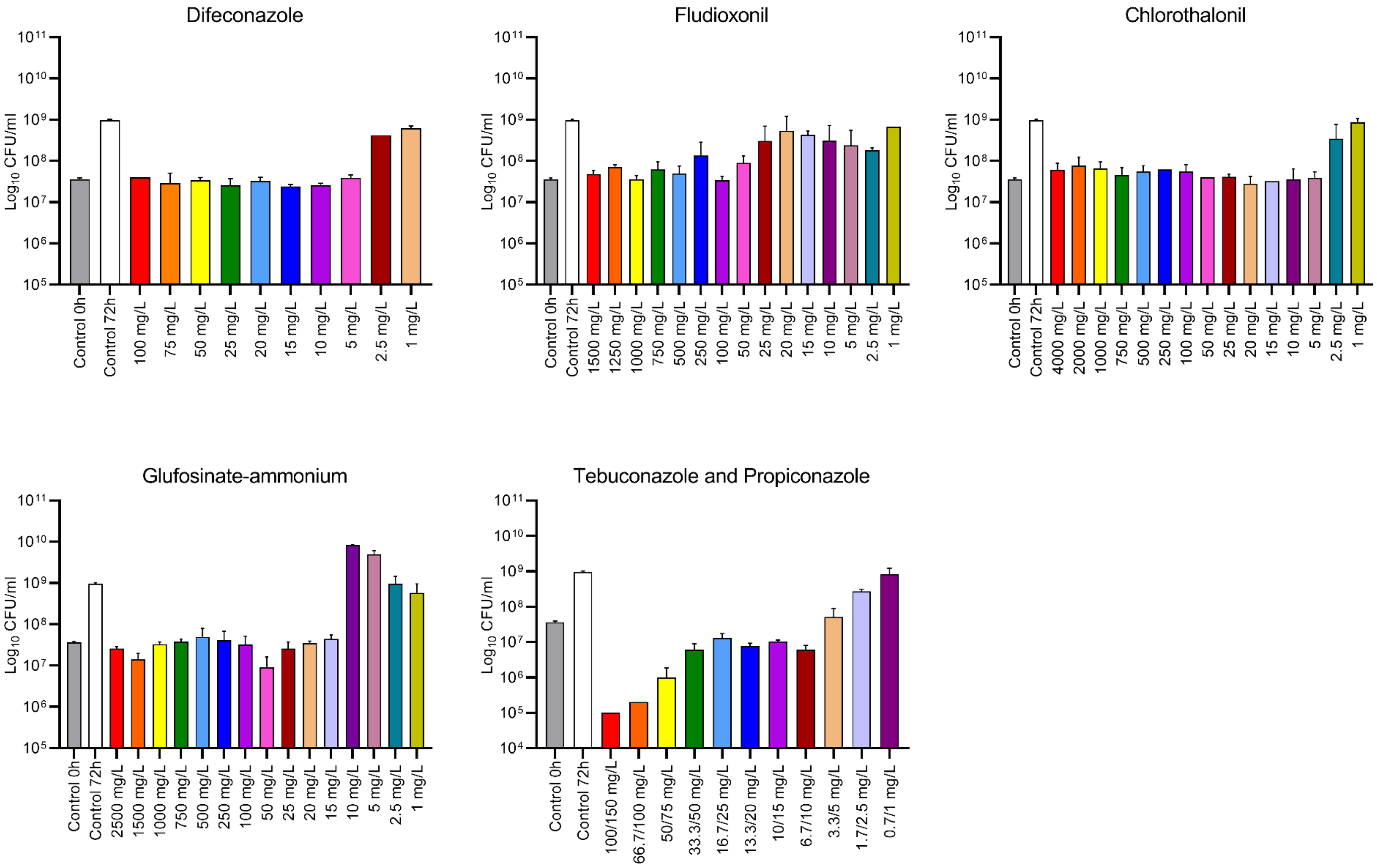

Figure S1. The survival of *N. mangyaensis* NH1 following exposure to pesticides. The assessment of bacterial viability was performed following a 72-hour incubation at 30°C in Mueller-Hinton medium, utilizing a range of concentrations for pesticides. Values are means ± standard deviations. All experiments were performed in duplicate.

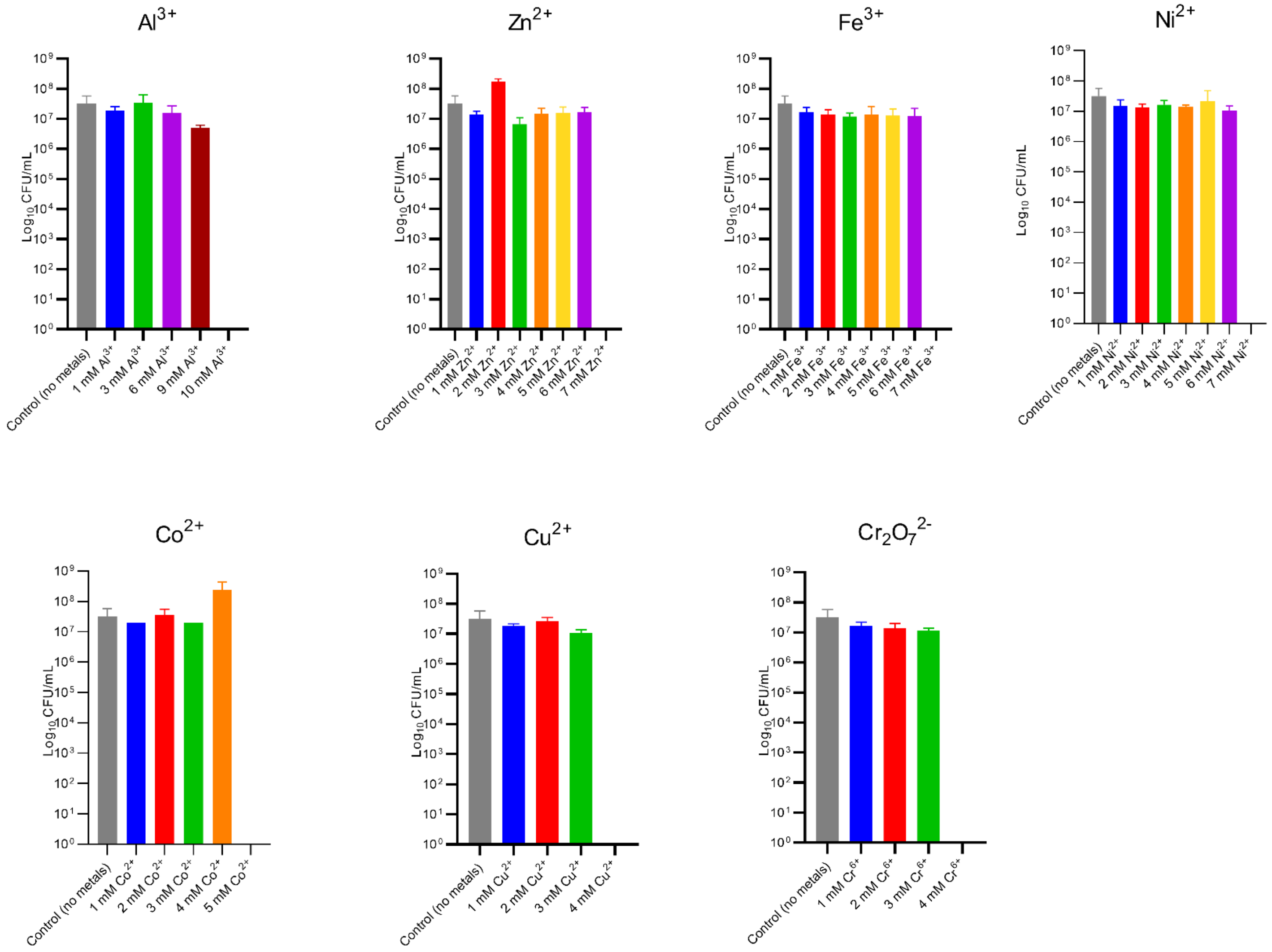

Figure S2. Determination of the minimum inhibitory concentrations (MIC) of heavy metals for *N. mangyaensis* NH1. Bacterial growth was assessed after 72-hour incubation at 30°C on Mueller-Hinton agar with a range of concentrations for heavy metals. Data represent the mean ± standard deviation from three independent experiments.

Table S2. *Nocardia mangyaensis* NH1 antibiotics susceptibility.

Class	Antibiotic (disk name)	Disk content (µg)	Clear zone (mm)	Susceptibility	Interpretation threshold (mm)*, **	
					S	R
Aminoglycosides	Neomycin	30	21.5±1.29	S	≥15	≤12
	Kanamycin	30	0	R	≥18	≤13
	Gentamicin†	120	27.5±1.00	-	-	-
	Gentamicin†	10	19.5±0.7	-	-	-
	Tobramycin††	10	18.5±0.7	R	≥18	<16
	Streptomycin †	10	0	R		
	Amikacin	30	24	S	≥17	<15
Macrolides	Erythromycin	15	16.0±1.82	I	≥22	<17
	Azithromycin†	15	16.25±1.25	-	-	-
Tetracyclines	Tetracycline	30	15.75±0.50	I	≥19	≤14
	Doxycycline	30	15.75±0.50	R	≥19	<17
Fluoroquinolones	Ciprofloxacin	5	21.5±1.29	I	≥25	<22
Cephalosporins	Ceftazidime	30	0	R	≥21	≤17
	Cefazolin†	30	0	R	-	-
Glycopeptide antibiotics	Vancomycin	30	15.5±1.0	R	≥17	-

Nitrofurans	Nitrofurantoin†	300	0	R	-	-

Footnotes: S, susceptible. I, Intermediate. R, resistant. -, No data.

All experiments were performed in triplicates.

*Interpretation threshold (mm) as described by Lebeaux, D., Bergeron, E., Berthet, J., Djadi-Prat, J., Mouniee, D., Boiron, P., Lortholary, O. and Rodriguez-Nava, V., 2019. Antibiotic susceptibility testing and species identification of *Nocardia* isolates: a retrospective analysis of data from a French expert laboratory, 2010–2015. Clinical Microbiology and Infection, 25(4), 489–495.

** Xu H, Xu R, Wang X, Liang Q, Zhang L, Liu J, Wei J, Lu Y and Yu D (2022) Co-infections of *Aeromonas veronii* and *Nocardia seriolae* in largemouth bass (*Micropterus salmoides*). Microb Pathog 173(Pt A), 105815. https://doi.org/10.1016/j.micpath.2022.105815.

†The criteria for disk diffusion susceptibility testing of *Nocardia* species using these antibiotics are not available.

†† Prudhomme C, Joannard B, Lina G, De Launay E, Dumitrescu O and Hodille E (2024) Drug susceptibility testing of *Nocardia* spp. using the disk diffusion method. Annals of Clinical Microbiology and Antimicrobials 23(1), 105. https://doi.org/10.1186/s12941-024-00768-2.

Table S3. Predicted genomic islands and virulence/resistance gene in the genome of *N. mangyaensis* NH1. The prediction was performed by integrating three different methods (IslandPath-DIMOB, SIGI-HMM, and IslandPick) by using the IslandViewer webserver.

Island start	Island end	Length	Method	Gene name	Gene ID	Locus	Gene start	Gene end	Strand	Product	External Annotations
2444097	2471497	27400	Predicted by at least one method	MDO3645649.1	smpB	Q3A91_01575	2444097	2444570	1	SsrA-binding protein SmpB	
2444097	2471497	27400	Predicted by at least one method	MDO3645650.1		Q3A91_01580	2444567	2445430	1	DMT family transporter	
2444097	2471497	27400	Predicted by at least one method	MDO3645651.1		Q3A91_01585	2445433	2446731	-1	ABC transporter substrate-binding protein	
2444097	2471497	27400	Predicted by at least one method	MDO3645652.1		Q3A91_01595	2447537	2448940	1	hypothetical protein	
2444097	2471497	27400	Predicted by at least one method	MDO3645653.1		Q3A91_01600	2448957	2450486	-1	tyrosine-type recombinase/integrase	
2444097	2471497	27400	Predicted by at least one method	MDO3645654.1		Q3A91_01605	2450483	2450806	-1	helix-turn-helix domain-containing protein	
2444097	2471497	27400	Predicted by at least one method	MDO3645655.1		Q3A91_01610	2450845	2451300	-1	recombinase family protein	
2444097	2471497	27400	Predicted by at least one method	MDO3645656.1		Q3A91_01615	2451297	2451953	-1	ImmA/IrrE family metallo-endopeptidase	
2444097	2471497	27400	Predicted by at least one method	MDO3645657.1		Q3A91_01620	2452046	2452408	-1	helix-turn-helix transcriptional regulator	
2444097	2471497	27400	Predicted by at least one method	MDO3645658.1		Q3A91_01625	2452550	2453185	-1	antirestriction protein ArdA	
2444097	2471497	27400	Predicted by at least one method	MDO3645659.1		Q3A91_01630	2453178	2453588	-1	hypothetical protein	
2444097	2471497	27400	Predicted by at least one method	MDO3645660.1		Q3A91_01635	2453695	2454072	-1	hypothetical protein	
2444097	2471497	27400	Predicted by at least one method	MDO3645661.1		Q3A91_01640	2454187	2454774	-1	MT-A70 family methyltransferase	
2444097	2471497	27400	Predicted by at least one method	MDO3645662.1		Q3A91_01645	2454764	2455771	-1	replication-relaxation family protein	
2444097	2471497	27400	Predicted by at least one method	MDO3645663.1		Q3A91_01650	2455768	2457951	-1	type IV secretory system conjugative DNA transfer family protein	
2444097	2471497	27400	Predicted by at least one method	MDO3645664.1		Q3A91_01655	2457951	2458196	-1	hypothetical protein	
2444097	2471497	27400	Predicted by at least one method	MDO3645665.1		Q3A91_01660	2458405	2458611	-1	hypothetical protein	

2444097	2471497	27400	Predicted by at least one method	MDO3645666.1		Q3A91_01665	2458614	2460119	-1	hypothetical protein	
2444097	2471497	27400	Predicted by at least one method	MDO3645667.1		Q3A91_01670	2460116	2461252	-1	OmpA family protein	
2444097	2471497	27400	Predicted by at least one method	MDO3645668.1		Q3A91_01675	2461368	2461907	-1	hypothetical protein	
2444097	2471497	27400	Predicted by at least one method	MDO3645669.1		Q3A91_01680	2462064	2463173	1	hypothetical protein	
2444097	2471497	27400	Predicted by at least one method	MDO3645670.1		Q3A91_01685	2463251	2464087	-1	hypothetical protein	
2444097	2471497	27400	Predicted by at least one method	MDO3645671.1		Q3A91_01690	2464257	2464493	1	hypothetical protein	
2444097	2471497	27400	Predicted by at least one method	MDO3645672.1		Q3A91_01695	2464649	2465761	1	hypothetical protein	
2444097	2471497	27400	Predicted by at least one method	MDO3645673.1		Q3A91_01700	2465730	2466560	-1	helix-turn-helix transcriptional regulator	
2444097	2471497	27400	Predicted by at least one method	MDO3645674.1		Q3A91_01705	2467017	2467919	1	TauD/TfdA family dioxygenase	
2444097	2471497	27400	Predicted by at least one method	MDO3645675.1		Q3A91_01710	2467962	2468705	1	alpha/beta fold hydrolase	
2444097	2471497	27400	Predicted by at least one method	MDO3645676.1		Q3A91_01715	2469887	2471497	1	hypothetical protein	
2444097	2471497	27400	Predicted by at least one method	MDO3645677.1		Q3A91_01720	2471494	2472261	1	ESX secretion-associated protein EspG	
2475831	2488219	12388	Predicted by at least one method	MDO3645684.1		Q3A91_01755	2475831	2476148	1	hypothetical protein	
2475831	2488219	12388	Predicted by at least one method	MDO3645685.1		Q3A91_01760	2476221	2477825	-1	SIR2 family protein	
2475831	2488219	12388	Predicted by at least one method	MDO3645686.1		Q3A91_01765	2477909	2479087	-1	hypothetical protein	

2475831	2488219	12388	Predicted by at least one method	MDO3645687.1		Q3A91_01770	2479452	2479712	1	helix-turn-helix transcriptional regulator	
2475831	2488219	12388	Predicted by at least one method	MDO3645688.1		Q3A91_01775	2480086	2480991	1	site-specific DNA-methyltransferase	
2475831	2488219	12388	Predicted by at least one method	MDO3645689.1		Q3A91_01780	2480984	2481646	-1	hypothetical protein	
2475831	2488219	12388	Predicted by at least one method	MDO3645690.1		Q3A91_01785	2482493	2483803	1	reverse transcriptase domain-containing protein	
2475831	2488219	12388	Predicted by at least one method			Q3A91_01790	2484286	2484446	1	IS5/IS1182 family transposase	
2475831	2488219	12388	Predicted by at least one method	MDO3645691.1		Q3A91_01795	2484500	2485714	-1	DUF4263 domain-containing protein	
2475831	2488219	12388	Predicted by at least one method	MDO3645692.1		Q3A91_01800	2485840	2486193	-1	hypothetical protein	
2475831	2488219	12388	Predicted by at least one method	MDO3645693.1		Q3A91_01805	2486154	2486579	-1	hypothetical protein	
2475831	2488219	12388	Predicted by at least one method	MDO3645694.1		Q3A91_01810	2487006	2487740	1	helix-turn-helix transcriptional regulator	
2475831	2488219	12388	Predicted by at least one method	MDO3645695.1		Q3A91_01815	2488007	2488219	-1	DUF5302 domain-containing protein	
3224360	3240363	16003	Predicted by at least one method	MDO3648959.1	mraY	Q3A91_18475	3224360	3225433	-1	phospho-N-acetylmuramoyl-pentapeptide-transferase	
3224360	3240363	16003	Predicted by at least one method	MDO3648958.1		Q3A91_18450	3226899	3227300	1	hypothetical protein	
3224360	3240363	16003	Predicted by at least one method	MDO3648957.1		Q3A91_18445	3227300	3227923	1	hypothetical protein	
3224360	3240363	16003	Predicted by at least one method	MDO3648956.1		Q3A91_18440	3227927	3233455	1	DUF6531 domain-containing protein	
3224360	3240363	16003	Predicted by at least one method	MDO3648955.1		Q3A91_18435	3233940	3235322	-1	IS1380 family transposase	

3224360	3240363	16003	Predicted by at least one method	MDO3648954.1		Q3A91_18430	3235441	3236487	-1	IS630 family transposase	
3224360	3240363	16003	Predicted by at least one method	MDO3646588.1		Q3A91_06375	3238145	3238261	1	IS5/IS1182 family transposase	
3224360	3240363	16003	Predicted by at least one method	MDO3646589.1		Q3A91_06380	3239219	3240363	-1	IS3 family transposase	
6607410	6650645	43235	Predicted by at least one method	MDO3651277.1		Q3A91_30425	6607410	6608165	-1	ESX secretion-associated protein EspG	
6607410	6650645	43235	Predicted by at least one method	MDO3651276.1		Q3A91_30420	6608162	6609499	-1	hypothetical protein	
6607410	6650645	43235	Predicted by at least one method	MDO3651275.1		Q3A91_30415	6609557	6609949	-1	hypothetical protein	
6607410	6650645	43235	Predicted by at least one method	MDO3651274.1		Q3A91_30410	6609981	6610532	-1	DUF3558 domain-containing protein	
6607410	6650645	43235	Predicted by at least one method	MDO3651273.1		Q3A91_30405	6610583	6611008	-1	HIT family protein	
6607410	6650645	43235	Predicted by at least one method	MDO3651272.1		Q3A91_30400	6611090	6611965	1	transposase	
6607410	6650645	43235	Predicted by at least one method	MDO3651422.1		Q3A91_31180	6614693	6615256	1	condensation domain-containing protein	
6607410	6650645	43235	Predicted by at least one method	MDO3651368.1		Q3A91_30905	6619687	6620348	-1	hypothetical protein	
6607410	6650645	43235	Predicted by at least one method	MDO3651445.1		Q3A91_31300	6622880	6623411	1	hypothetical protein	
6607410	6650645	43235	Predicted by at least one method	MDO3651453.1		Q3A91_31345	6624412	6624758	-1	hypothetical protein	
6607410	6650645	43235	Predicted by at least one method	MDO3651259.1		Q3A91_30315	6626578	6626691	1	LuxR C-terminal-related transcriptional reg-ulator	
6607410	6650645	43235	Predicted by at least one method	MDO3651260.1		Q3A91_30320	6626747	6627472	-1	DJ-1/PfpI family protein	

6607410	6650645	43235	Predicted by at least one method	MDO3651261.1		Q3A91_30325	6627549	6628001	-1	nitroreductase/quinone reductase family protein	
6607410	6650645	43235	Predicted by at least one method	MDO3651262.1		Q3A91_30330	6627994	6628434	-1	hypothetical protein	
6607410	6650645	43235	Predicted by at least one method	MDO3651263.1		Q3A91_30335	6628597	6629436	-1	alkylmercury lyase family protein	
6607410	6650645	43235	Predicted by at least one method	MDO3651264.1		Q3A91_30340	6629780	6629992	-1	hypothetical protein	
6607410	6650645	43235	Predicted by at least one method	MDO3651265.1		Q3A91_30345	6630284	6631222	1	TIGR03619 family F420-dependent LLM class oxidoreductase	
6607410	6650645	43235	Predicted by at least one method	MDO3651266.1		Q3A91_30350	6631333	6631695	-1	alpha/beta hydrolase	
6607410	6650645	43235	Predicted by at least one method	MDO3651267.1		Q3A91_30355	6631728	6632102	-1	alpha/beta fold hydrolase	
6607410	6650645	43235	Predicted by at least one method			Q3A91_30360	6632297	6632437	1	3-hydroxyacyl-CoA dehydrogenase	
6607410	6650645	43235	Predicted by at least one method	MDO3651268.1		Q3A91_30365	6632470	6633153	-1	GntR family transcriptional regulator	
6607410	6650645	43235	Predicted by at least one method	MDO3651376.1		Q3A91_30945	6634507	6634833	-1	hypothetical protein	
6607410	6650645	43235	Predicted by at least one method	MDO3651370.1		Q3A91_30915	6636151	6636598	-1	hypothetical protein	
6607410	6650645	43235	Predicted by at least one method	MDO3651371.1		Q3A91_30920	6636627	6636821	-1	hypothetical protein	
6607410	6650645	43235	Predicted by at least one method	MDO3651362.1		Q3A91_30875	6639848	6640443	-1	integrase	
6607410	6650645	43235	Predicted by at least one method	MDO3651373.1		Q3A91_30930	6641566	6642122	1	hypothetical protein	
6607410	6650645	43235	Predicted by at least one method	MDO3651156.1		Q3A91_29775	6649009	6649485	-1	hypothetical protein	

6607410	6650645	43235	Predicted by at least one method	MDO3651157.1		Q3A91_29780	6649526	6649915	-1	hypothetical protein	
6607410	6650645	43235	Predicted by at least one method	MDO3651158.1		Q3A91_29785	6650361	6650645	-1	hypothetical protein	
6669361	6938808	269447	Predicted by at least one method	MDO3651183.1		Q3A91_29915	6668240	6669364	1	hypothetical protein	
6669361	6938808	269447	Predicted by at least one method	MDO3651184.1		Q3A91_29920	6669361	6669681	1	hypothetical protein	
6669361	6938808	269447	Predicted by at least one method	MDO3651185.1		Q3A91_29925	6669678	6670466	1	DUF2637 domain-containing protein	
6669361	6938808	269447	Predicted by at least one method	MDO3651186.1		Q3A91_29930	6670549	6671004	1	hypothetical protein	
6669361	6938808	269447	Predicted by at least one method	MDO3651441.1		Q3A91_31280	6672006	6672226	-1	hypothetical protein	
6669361	6938808	269447	Predicted by at least one method	MDO3651442.1		Q3A91_31285	6672289	6672540	1	hypothetical protein	
6669361	6938808	269447	Predicted by at least one method	MDO3651316.1		Q3A91_30630	6673608	6674495	-1	helix-turn-helix domain-containing protein	
6669361	6938808	269447	Predicted by at least one method	MDO3651295.1		Q3A91_30520	6675765	6677462	1	endonuclease domain-containing protein	
6669361	6938808	269447	Predicted by at least one method	MDO3651424.1		Q3A91_31190	6678588	6678977	-1	class I tRNA ligase family protein	
6669361	6938808	269447	Predicted by at least one method	MDO3651289.1		Q3A91_30485	6680584	6681548	1	hypothetical protein	
6669361	6938808	269447	Predicted by at least one method	MDO3651290.1		Q3A91_30490	6681961	6682613	1	major capsid protein	
6669361	6938808	269447	Predicted by at least one method	MDO3651326.1		Q3A91_30690	6683678	6684379	1	hypothetical protein	
6669361	6938808	269447	Predicted by at least one method	MDO3651421.1		Q3A91_31175	6685589	6686100	-1	DUF1524 domain-containing protein	

6669361	6938808	269447	Predicted by at least one method	MDO3651360.1		Q3A91_30865	6687384	6687875	1	ATP-dependent Clp protease proteolytic subunit	
6669361	6938808	269447	Predicted by at least one method	MDO3651410.1		Q3A91_31120	6688876	6689457	1	hypothetical protein	
6669361	6938808	269447	Predicted by at least one method			Q3A91_31350	6690458	6690928	-1	AMP-binding protein	
6669361	6938808	269447	Predicted by at least one method	MDO3651390.1		Q3A91_31020	6691975	6692587	1	hypothetical protein	
6669361	6938808	269447	Predicted by at least one method	MDO3651347.1		Q3A91_30800	6693762	6693959	1	hypothetical protein	
6669361	6938808	269447	Predicted by at least one method	MDO3651348.1		Q3A91_30805	6694055	6694356	-1	hypothetical protein	
6669361	6938808	269447	Predicted by at least one method	MDO3651432.1		Q3A91_31235	6695357	6695869	-1	hypothetical protein	
6669361	6938808	269447	Predicted by at least one method	MDO3651429.1		Q3A91_31220	6696870	6697423	1	AAA family ATPase	
6669361	6938808	269447	Predicted by at least one method	MDO3651351.1		Q3A91_30820	6700319	6700546	-1	hypothetical protein	
6669361	6938808	269447	Predicted by at least one method	MDO3651352.1		Q3A91_30825	6700546	6700734	-1	hypothetical protein	
6669361	6938808	269447	Predicted by at least one method	MDO3651246.1		Q3A91_30240	6702525	6703028	1	hypothetical protein	
6669361	6938808	269447	Predicted by at least one method	MDO3651247.1		Q3A91_30245	6703164	6703553	1	hypothetical protein	
6669361	6938808	269447	Predicted by at least one method	MDO3651248.1		Q3A91_30250	6703550	6704140	1	DUF3558 domain-containing protein	
6669361	6938808	269447	Predicted by at least one method	MDO3651249.1		Q3A91_30255	6704137	6704676	-1	DUF3558 family protein	
6669361	6938808	269447	Predicted by at least one method	MDO3651250.1		Q3A91_30260	6704694	6707195	-1	hypothetical protein	

6669361	6938808	269447	Predicted by at least one method	MDO3651251.1		Q3A91_30265	6707195	6707503	-1	hypothetical protein	
6669361	6938808	269447	Predicted by at least one method	MDO3651252.1		Q3A91_30270	6707696	6709411	1	hypothetical protein	
6669361	6938808	269447	Predicted by at least one method	MDO3651253.1		Q3A91_30275	6709420	6710160	1	ESX secretion-associated protein EspG	
6669361	6938808	269447	Predicted by at least one method			Q3A91_30280	6710356	6712509	-1	damage-inducible protein	
6669361	6938808	269447	Predicted by at least one method	MDO3651412.1		Q3A91_31130	6713638	6713791	-1	elongation factor Ts	
6669361	6938808	269447	Predicted by at least one method	MDO3651413.1	rpsB	Q3A91_31135	6713793	6714215	-1	30S ribosomal protein S2	
6669361	6938808	269447	Predicted by at least one method	MDO3651457.1		Q3A91_31370	6715216	6715730	-1	hypothetical protein	
6669361	6938808	269447	Predicted by at least one method	MDO3651345.1		Q3A91_30790	6716731	6716996	-1	cation diffusion facilitator family transporter	
6669361	6938808	269447	Predicted by at least one method	MDO3651346.1		Q3A91_30795	6716996	6717513	-1	carbamoyl phosphate synthase small subunit	
6669361	6938808	269447	Predicted by at least one method	MDO3651339.1		Q3A91_30755	6718514	6718857	1	hypothetical protein	
6669361	6938808	269447	Predicted by at least one method	MDO3651340.1		Q3A91_30760	6718926	6719261	1	WXG100 family type VII secretion target	
6669361	6938808	269447	Predicted by at least one method	MDO3651341.1		Q3A91_30765	6719240	6719356	-1	IS5/IS1182 family transposase	
6669361	6938808	269447	Predicted by at least one method	MDO3651305.1		Q3A91_30575	6720357	6720775	1	hypothetical protein	
6669361	6938808	269447	Predicted by at least one method	MDO3651306.1		Q3A91_30580	6720911	6721588	1	hypothetical protein	
6669361	6938808	269447	Predicted by at least one method	MDO3651396.1		Q3A91_31050	6722999	6723235	-1	hypothetical protein	

6669361	6938808	269447	Predicted by at least one method	MDO3651454.1		Q3A91_31355	6724619	6724955	-1	hypothetical protein	
6669361	6938808	269447	Predicted by at least one method	MDO3651388.1		Q3A91_31010	6725956	6726463	-1	hypothetical protein	
6669361	6938808	269447	Predicted by at least one method	MDO3651406.1		Q3A91_31100	6727593	6728162	1	Mur ligase family protein	
6669361	6938808	269447	Predicted by at least one method	MDO3651335.1		Q3A91_30735	6729163	6729500	1	type I pantothenate kinase	
6669361	6938808	269447	Predicted by at least one method	MDO3651336.1		Q3A91_30740	6729545	6730056	-1	hypothetical protein	
6669361	6938808	269447	Predicted by at least one method	MDO3651425.1	thyX	Q3A91_31195	6731057	6731615	-1	FAD-dependent thymidylate synthase	
6669361	6938808	269447	Predicted by at least one method	MDO3651383.1		Q3A91_30985	6732616	6733120	1	hypothetical protein	
6669361	6938808	269447	Predicted by at least one method	MDO3651308.1		Q3A91_30590	6734387	6734611	1	hypothetical protein	
6669361	6938808	269447	Predicted by at least one method	MDO3651309.1		Q3A91_30595	6734613	6735218	1	hypothetical protein	
6669361	6938808	269447	Predicted by at least one method	MDO3651310.1		Q3A91_30600	6735231	6735619	1	hypothetical protein	
6669361	6938808	269447	Predicted by at least one method	MDO3651458.1		Q3A91_31375	6736620	6737133	-1	pyridoxal-phosphate dependent enzyme	
6669361	6938808	269447	Predicted by at least one method	MDO3651329.1		Q3A91_30705	6738209	6738487	-1	hypothetical protein	
6669361	6938808	269447	Predicted by at least one method	MDO3651330.1		Q3A91_30710	6738480	6739097	-1	hypothetical protein	
6669361	6938808	269447	Predicted by at least one method	MDO3651416.1		Q3A91_31150	6740442	6740669	-1	hypothetical protein	
6669361	6938808	269447	Predicted by at least one method	MDO3651354.1		Q3A91_30835	6741670	6742407	1	Eco57I restriction-modification methylase domain-containing protein	

6669361	6938808	269447	Predicted by at least one method	MDO3651358.1	infC	Q3A91_30855	6743408	6743970	-1	translation initiation factor IF-3	
6669361	6938808	269447	Predicted by at least one method	MDO3651398.1		Q3A91_31060	6745151	6745724	1	class I tRNA ligase family protein	
6669361	6938808	269447	Predicted by at least one method	MDO3651334.1		Q3A91_30730	6749046	6749822	1	transposase family protein	
6669361	6938808	269447	Predicted by at least one method	MDO3651303.1		Q3A91_30565	6750833	6751502	1	hypothetical protein	
6669361	6938808	269447	Predicted by at least one method	MDO3651304.1		Q3A91_30570	6751502	6752332	1	hypothetical protein	
6669361	6938808	269447	Predicted by at least one method	MDO3651342.1		Q3A91_30770	6753472	6754160	-1	hypothetical protein	
6669361	6938808	269447	Predicted by at least one method	MDO3651437.1		Q3A91_31260	6755161	6755700	-1	preprotein translocase subunit SecA	
6669361	6938808	269447	Predicted by at least one method	MDO3651393.1		Q3A91_31035	6756701	6757056	-1	laccase domain-containing protein	
6669361	6938808	269447	Predicted by at least one method	MDO3651394.1		Q3A91_31040	6757074	6757303	1	hypothetical protein	
6669361	6938808	269447	Predicted by at least one method	MDO3651462.1		Q3A91_31395	6758394	6758807	1	DeoR/GlpR family DNA-binding transcrip-tion regulator	
6669361	6938808	269447	Predicted by at least one method	MDO3651369.1		Q3A91_30910	6759902	6760485	1	hypothetical protein	
6669361	6938808	269447	Predicted by at least one method	MDO3651325.1		Q3A91_30685	6761757	6762395	-1	ISAs1 family transposase	
6669361	6938808	269447	Predicted by at least one method	MDO3651382.1		Q3A91_30980	6763396	6763764	-1	major capsid protein	
6669361	6938808	269447	Predicted by at least one method	MDO3651402.1		Q3A91_31080	6765027	6765302	1	hypothetical protein	
6669361	6938808	269447	Predicted by at least one method	MDO3651403.1		Q3A91_31085	6765302	6765614	1	hypothetical protein	

6669361	6938808	269447	Predicted by at least one method	MDO3651460.1		Q3A91_31385	6766615	6766963	1	cytochrome c	
6669361	6938808	269447	Predicted by at least one method	MDO3651401.1		Q3A91_31075	6768125	6768714	1	helicase C-terminal domain-containing protein	
6669361	6938808	269447	Predicted by at least one method	MDO3651419.1		Q3A91_31165	6769825	6770190	1	hypothetical protein	
6669361	6938808	269447	Predicted by at least one method	MDO3651446.1		Q3A91_31310	6771251	6771395	-1	DoxX family membrane protein	
6669361	6938808	269447	Predicted by at least one method	MDO3651447.1		Q3A91_31315	6771500	6771782	-1	LuxR C-terminal-related transcriptional regulator	
6669361	6938808	269447	Predicted by at least one method	MDO3651399.1		Q3A91_31065	6772978	6773196	1	hypothetical protein	
6669361	6938808	269447	Predicted by at least one method	MDO3651400.1		Q3A91_31070	6773193	6773373	1	hypothetical protein	
6669361	6938808	269447	Predicted by at least one method	MDO3651452.1		Q3A91_31340	6774374	6774898	-1	5'/3'-nucleotidase SurE	
6669361	6938808	269447	Predicted by at least one method	MDO3651320.1		Q3A91_30650	6775899	6776965	1	hypothetical protein	
6669361	6938808	269447	Predicted by at least one method	MDO3651286.1		Q3A91_30470	6777966	6779516	1	hypothetical protein	
6669361	6938808	269447	Predicted by at least one method	MDO3651287.1		Q3A91_30475	6779506	6780033	-1	hypothetical protein	
6669361	6938808	269447	Predicted by at least one method	MDO3651288.1		Q3A91_30480	6779957	6780391	-1	hypothetical protein	
6669361	6938808	269447	Predicted by at least one method	MDO3651377.1		Q3A91_30950	6781695	6782290	1	transposase	
6669361	6938808	269447	Predicted by at least one method	MDO3651291.1		Q3A91_30495	6783291	6783491	1	hypothetical protein	
6669361	6938808	269447	Predicted by at least one method	MDO3651292.1		Q3A91_30500	6784233	6785257	-1	phage/plasmid replication protein, II/X family	

6669361	6938808	269447	Predicted by at least one method	MDO3651420.1		Q3A91_31170	6786258	6786705	-1	polyribonucleotide nucleotidyltransferase	
6669361	6938808	269447	Predicted by at least one method	MDO3651455.1		Q3A91_31360	6787827	6788341	-1	DUF839 domain-containing protein	
6669361	6938808	269447	Predicted by at least one method	MDO3651343.1		Q3A91_30780	6791490	6791699	-1	acetyl-CoA carboxylase carboxyl transferase subunit beta	
6669361	6938808	269447	Predicted by at least one method	MDO3651380.1		Q3A91_30970	6792700	6793227	-1	7-cyano-7-deazaguanine synthase	
6669361	6938808	269447	Predicted by at least one method	MDO3651450.1		Q3A91_31330	6794333	6794706	-1	hypothetical protein	
6669361	6938808	269447	Predicted by at least one method	MDO3651385.1		Q3A91_30995	6795861	6796482	-1	hypothetical protein	
6669361	6938808	269447	Predicted by at least one method	MDO3651426.1		Q3A91_31200	6797535	6798040	-1	NUMOD4 domain-containing protein	
6669361	6938808	269447	Predicted by at least one method	MDO3651444.1		Q3A91_31295	6799319	6799574	-1	hypothetical protein	
6669361	6938808	269447	Predicted by at least one method	MDO3651313.1		Q3A91_30615	6800593	6801767	1	IS3 family transposase	
6669361	6938808	269447	Predicted by at least one method	MDO3651365.1		Q3A91_30890	6802810	6803501	1	NAD-glutamate dehydrogenase	
6669361	6938808	269447	Predicted by at least one method	MDO3651461.1		Q3A91_31390	6804502	6804982	1	hypothetical protein	
6669361	6938808	269447	Predicted by at least one method	MDO3651312.1		Q3A91_30610	6805983	6807229	-1	hypothetical protein	
6669361	6938808	269447	Predicted by at least one method	MDO3651404.1		Q3A91_31090	6808230	6808412	1	HU family DNA-binding protein	
6669361	6938808	269447	Predicted by at least one method	MDO3651405.1		Q3A91_31095	6808441	6808817	-1	DUF3768 domain-containing protein	
6669361	6938808	269447	Predicted by at least one method	MDO3651397.1		Q3A91_31055	6814237	6814831	-1	tripartite tricarboxylate transporter substrate-binding protein	

6669361	6938808	269447	Predicted by at least one method	MDO3651349.1		Q3A91_30810	6815832	6816250	1	NCS2 family permease	
6669361	6938808	269447	Predicted by at least one method	MDO3651350.1		Q3A91_30815	6816225	6816594	-1	hypothetical protein	
6669361	6938808	269447	Predicted by at least one method	MDO3651433.1		Q3A91_31240	6819465	6819647	1	hypothetical protein	
6669361	6938808	269447	Predicted by at least one method	MDO3651322.1		Q3A91_30660	6822412	6822975	1	hypothetical protein	
6669361	6938808	269447	Predicted by at least one method	MDO3651363.1		Q3A91_30880	6824321	6825034	-1	hypothetical protein	
6669361	6938808	269447	Predicted by at least one method	MDO3651323.1		Q3A91_30665	6826035	6826858	1	transposase	
6669361	6938808	269447	Predicted by at least one method	MDO3651364.1		Q3A91_30885	6828370	6828765	-1	hypothetical protein	
6669361	6938808	269447	Predicted by at least one method			Q3A91_30370	6831709	6831801	-1	hypothetical protein	
6669361	6938808	269447	Predicted by at least one method	MDO3651269.1		Q3A91_30375	6832610	6832804	1	hypothetical protein	
6669361	6938808	269447	Predicted by at least one method	MDO3651270.1	aac(3)-IVa	Q3A91_30380	6832870	6833646	-1	aminoglycoside N-acetyltransferase AAC(3)-IVa	
6669361	6938808	269447	Predicted by at least one method			Q3A91_30385	6833760	6833852	1	IS6 family transposase	
6669361	6938808	269447	Predicted by at least one method	MDO3651271.1	cas9	Q3A91_30390	6834760	6837807	-1	type II CRISPR RNA-guided endonuclease Cas9	
6669361	6938808	269447	Predicted by at least one method			Q3A91_30395	6837954	6838130	-1	CII family transcriptional regulator	
6669361	6938808	269447	Predicted by at least one method	MDO3651327.1		Q3A91_30695	6839260	6839562	-1	major capsid protein	
6669361	6938808	269447	Predicted by at least one method	MDO3651328.1		Q3A91_30700	6839854	6840231	-1	hypothetical protein	

6669361	6938808	269447	Predicted by at least one method	MDO3651324.1		Q3A91_30680	6842787	6843198	-1	hypothetical protein	
6669361	6938808	269447	Predicted by at least one method	MDO3651392.1		Q3A91_31030	6844806	6845045	-1	hypothetical protein	
6669361	6938808	269447	Predicted by at least one method	MDO3651379.1		Q3A91_30965	6846862	6847045	-1	hypothetical protein	
6669361	6938808	269447	Predicted by at least one method	MDO3651389.1		Q3A91_31015	6848046	6848665	-1	ATP-binding cassette domain-containing protein	
6669361	6938808	269447	Predicted by at least one method	MDO3651344.1		Q3A91_30785	6849666	6850426	-1	hypothetical protein	
6669361	6938808	269447	Predicted by at least one method	MDO3651333.1		Q3A91_30725	6851751	6852311	1	zonular occludens toxin domain-containing protein	
6669361	6938808	269447	Predicted by at least one method	MDO3651438.1		Q3A91_31265	6853407	6853946	-1	hypothetical protein	
6669361	6938808	269447	Predicted by at least one method	MDO3651361.1		Q3A91_30870	6854947	6855666	-1	kelch repeat-containing protein	
6669361	6938808	269447	Predicted by at least one method	MDO3651366.1		Q3A91_30895	6856744	6856956	1	hypothetical protein	
6669361	6938808	269447	Predicted by at least one method	MDO3651367.1		Q3A91_30900	6856996	6857349	1	hypothetical protein	
6669361	6938808	269447	Predicted by at least one method	MDO3651357.1		Q3A91_30850	6858351	6859078	1	polysaccharide biosynthesis/export family protein	
6669361	6938808	269447	Predicted by at least one method	MDO3651319.1		Q3A91_30645	6860079	6861165	-1	hypothetical protein	
6669361	6938808	269447	Predicted by at least one method	MDO3651296.1		Q3A91_30525	6862343	6863005	-1	hypothetical protein	
6669361	6938808	269447	Predicted by at least one method	MDO3651297.1		Q3A91_30530	6863062	6863999	-1	major capsid protein	
6669361	6938808	269447	Predicted by at least one method	MDO3651448.1		Q3A91_31320	6865000	6865524	-1	DUF4347 domain-containing protein	

6669361	6938808	269447	Predicted by at least one method	MDO3651298.1		Q3A91_30540	6866531	6866838	-1	hypothetical protein	
6669361	6938808	269447	Predicted by at least one method	MDO3651299.1		Q3A91_30545	6867675	6868210	-1	hypothetical protein	
6669361	6938808	269447	Predicted by at least one method	MDO3651338.1		Q3A91_30750	6869359	6870055	-1	transposase	
6669361	6938808	269447	Predicted by at least one method	MDO3651281.1		Q3A91_30445	6871875	6872633	1	hypothetical protein	
6669361	6938808	269447	Predicted by at least one method	MDO3651282.1		Q3A91_30450	6872807	6873079	1	hypothetical protein	
6669361	6938808	269447	Predicted by at least one method	MDO3651283.1		Q3A91_30455	6873780	6874221	1	hypothetical protein	
6669361	6938808	269447	Predicted by at least one method	MDO3651293.1		Q3A91_30505	6875222	6876674	1	hypothetical protein	
6669361	6938808	269447	Predicted by at least one method	MDO3651294.1		Q3A91_30510	6876846	6877153	-1	group II intron reverse transcriptase/matu-rase	
6669361	6938808	269447	Predicted by at least one method	MDO3651435.1		Q3A91_31250	6878154	6878654	-1	hypothetical protein	
6669361	6938808	269447	Predicted by at least one method	MDO3651355.1		Q3A91_30840	6879696	6879896	-1	hypothetical protein	
6669361	6938808	269447	Predicted by at least one method	MDO3651356.1		Q3A91_30845	6879960	6880423	-1	hypothetical protein	
6669361	6938808	269447	Predicted by at least one method	MDO3651436.1	folB	Q3A91_31255	6881490	6881852	1	dihydroneopterin aldolase	
6669361	6938808	269447	Predicted by at least one method	MDO3651417.1		Q3A91_31155	6882965	6883257	1	DUF1996 domain-containing protein	
6669361	6938808	269447	Predicted by at least one method	MDO3651418.1		Q3A91_31160	6883289	6883535	-1	RNHCP domain-containing protein	
6669361	6938808	269447	Predicted by at least one method	MDO3651317.1		Q3A91_30635	6884536	6884924	1	hypothetical protein	

6669361	6938808	269447	Predicted by at least one method	MDO3651318.1		Q3A91_30640	6885217	6885694	1	zonular occludens toxin domain-containing protein	
6669361	6938808	269447	Predicted by at least one method	MDO3651378.1		Q3A91_30955	6886807	6887333	-1	hypothetical protein	
6669361	6938808	269447	Predicted by at least one method	MDO3651443.1		Q3A91_31290	6888334	6888867	1	hypothetical protein	
6669361	6938808	269447	Predicted by at least one method	MDO3651434.1		Q3A91_31245	6889868	6890413	1	SDR family oxidoreductase	
6669361	6938808	269447	Predicted by at least one method	MDO3651381.1		Q3A91_30975	6891470	6891883	1	reverse transcriptase domain-containing protein	
6669361	6938808	269447	Predicted by at least one method	MDO3651411.1	rpsP	Q3A91_31125	6893171	6893422	1	30S ribosomal protein S16	
6669361	6938808	269447	Predicted by at least one method	MDO3651409.1		Q3A91_31115	6896366	6896729	1	Fe-Mn family superoxide dismutase	
6669361	6938808	269447	Predicted by at least one method	MDO3651386.1	rpsD	Q3A91_31000	6897951	6898465	-1	30S ribosomal protein S4	
6669361	6938808	269447	Predicted by at least one method	MDO3651387.1	rpsK	Q3A91_31005	6898469	6898571	-1	30S ribosomal protein S11	
6669361	6938808	269447	Predicted by at least one method	MDO3651427.1		Q3A91_31205	6899572	6899756	1	hypothetical protein	
6669361	6938808	269447	Predicted by at least one method	MDO3651428.1		Q3A91_31210	6899731	6900129	1	hypothetical protein	
6669361	6938808	269447	Predicted by at least one method	MDO3651391.1		Q3A91_31025	6901269	6901735	1	transposase family protein	
6669361	6938808	269447	Predicted by at least one method	MDO3651284.1		Q3A91_30460	6902736	6904200	1	hypothetical protein	
6669361	6938808	269447	Predicted by at least one method	MDO3651285.1		Q3A91_30465	6904464	6905656	-1	hypothetical protein	
6669361	6938808	269447	Predicted by at least one method	MDO3651459.1		Q3A91_31380	6906836	6907167	1	hypothetical protein	

6669361	6938808	269447	Predicted by at least one method	MDO3651374.1		Q3A91_30935	6908168	6908524	1	hypothetical protein	
6669361	6938808	269447	Predicted by at least one method	MDO3651375.1		Q3A91_30940	6908521	6908814	1	DUF2791 family P-loop domain-containing protein	
6669361	6938808	269447	Predicted by at least one method	MDO3651301.1		Q3A91_30555	6909815	6910150	-1	hypothetical protein	
6669361	6938808	269447	Predicted by at least one method	MDO3651302.1		Q3A91_30560	6910386	6911330	-1	hypothetical protein	
6669361	6938808	269447	Predicted by at least one method	MDO3651307.1		Q3A91_30585	6912380	6913573	-1	IS110 family transposase	
6669361	6938808	269447	Predicted by at least one method	MDO3651415.1		Q3A91_31145	6915015	6915372	1	DeoR family transcriptional regulator	
6669361	6938808	269447	Predicted by at least one method	MDO3651353.1		Q3A91_30830	6916373	6917110	-1	Rieske 2Fe-2S domain-containing protein	
6669361	6938808	269447	Predicted by at least one method	MDO3651337.1		Q3A91_30745	6918295	6918983	1	hypothetical protein	
6669361	6938808	269447	Predicted by at least one method	MDO3651395.1		Q3A91_31045	6919984	6920586	1	hypothetical protein	
6669361	6938808	269447	Predicted by at least one method	MDO3651449.1		Q3A91_31325	6921587	6921769	1	hypothetical protein	
6669361	6938808	269447	Predicted by at least one method	MDO3651407.1		Q3A91_31105	6923117	6923304	-1	hypothetical protein	
6669361	6938808	269447	Predicted by at least one method	MDO3651408.1		Q3A91_31110	6923372	6923701	-1	hypothetical protein	
6669361	6938808	269447	Predicted by at least one method	MDO3651414.1		Q3A91_31140	6924702	6925190	1	hypothetical protein	
6669361	6938808	269447	Predicted by at least one method	MDO3651321.1		Q3A91_30655	6926275	6926765	1	AAA family ATPase	
6669361	6938808	269447	Predicted by at least one method	MDO3651315.1		Q3A91_30625	6929213	6929525	1	hypothetical protein	

6669361	6938808	269447	Predicted by at least one method	MDO3651456.1		Q3A91_31365	6930526	6931040	-1	hypothetical protein	
6669361	6938808	269447	Predicted by at least one method	MDO3651423.1		Q3A91_31185	6933654	6933861	-1	hypothetical protein	
6669361	6938808	269447	Predicted by at least one method	MDO3651430.1		Q3A91_31225	6935215	6935481	1	hypothetical protein	
6669361	6938808	269447	Predicted by at least one method	MDO3651431.1		Q3A91_31230	6935456	6935766	1	tRNA pseudouridine(55) synthase TruB	
6669361	6938808	269447	Predicted by at least one method	MDO3651331.1		Q3A91_30715	6936840	6937226	-1	hypothetical protein	
6669361	6938808	269447	Predicted by at least one method	MDO3651332.1		Q3A91_30720	6937261	6937709	-1	nicotinate phosphoribosyltransferase	
6669361	6938808	269447	Predicted by at least one method	MDO3651439.1		Q3A91_31270	6938710	6938808	1	ABC transporter permease	

Table S4 The summary of information on CRISPR arrays and *cas* gene clusters in the genome of *N. mangyaensis* NH1

Element	CRISPR Id /Cas Type	Start	End	Spacer / Gene	Repeat consensus / cas genes	Direction	Evidence Level
CRISPR	JAUMIP010000011_1_Nocardia_mangyaen-sis_strain_NH1_NODE_11_length_194708_cov_201_691434__whole_ge-nome_shotgun_sequence_1	7494	7588	1	CGGCTTCCCGGATTCCGGGTCGG	ND	1
CRISPR	JAUMIP010000013_1_Nocardia_mangyaen-sis_strain_NH1_NODE_13_length_164736_cov_210_933406__whole_ge-nome_shotgun_sequence_1	85	282	2	TGGGCATGAAGGGTGCCGCGAAGGCACCCGG CG	ND	1
CRISPR	JAUMIP010000016_1_Nocardia_mangyaen-sis_strain_NH1_NODE_16_length_143822_cov_215_544027__whole_ge-nome_shotgun_sequence_1	13725 8	13737 5	1	TGCGTAACCACGGAGGGGCAGGGAGTGGCGC GAATTCAACGC	ND	1
CRISPR	JAUMIP010000001_1_Nocardia_mangyaen-sis_strain_NH1_NODE_1_length_886673_cov_205_478458__whole_ge-nome_shotgun_sequence_1	12884 9	12894 8	1	GCGCATGGCCGGAGCGAATGCGGAGGTTTCG CC	ND	1
CRISPR	JAUMIP010000028_1_Nocardia_mangyaen-sis_strain_NH1_NODE_28_length_59890_cov_212_003782__whole_ge-nome_shotgun_sequence_1	36632	36724	1	GGGCCTCACACCCGAAGGGCGATACTG	ND	1
CRISPR	JAUMIP010000038_1_Nocardia_mangyaen-sis_strain_NH1_NODE_38_length_23495_cov_1085_410604__whole_ge-nome_shotgun_sequence_1	12329	12435	1	TGATGCAGTAGGAAACCCGAACCAT	ND	1
CRISPR	JAUMIP010000003_1_Nocardia_mangyaen-sis_strain_NH1_NODE_3_length_458888_cov_204_407408__whole_ge-nome_shotgun_sequence_1	67856	67927	1	CCAGATCGAGGCGGCCGAGAACC	ND	1
CRISPR	JAUMIP010000049_1_Nocardia_mangyaen-sis_strain_NH1_NODE_49_length_3166_cov_1_142152__whole_ge-nome_shotgun_sequence_1	68	278	4	GCGGGGGGGGGGGGGGGGGGGG	ND	2
CRISPR	JAUMIP010000005_1_Nocardia_mangyaen-sis_strain_NH1_NODE_5_length_377693_cov_215_669403__whole_ge-nome_shotgun_sequence_1	85320	85430	1	CCACCACCAACACCCGCCACCTCCCGC	ND	1
CRISPR	JAUMIP010000008_1_Nocardia_mangyaen-sis_strain_NH1_NODE_8_length_283780_cov_203_211244__whole_ge-nome_shotgun_sequence_1	469	681	2	CGATCGCGGTGGTTACCAGCGTCGTGACGATG CCGGCCG	ND	1
Cas cluster	JAUMIP010000046_1_Nocardia_mangyaen-sis_strain_NH1_NODE_46_length_6814_cov_8_610139__whole_ge-nome_shotgun_sequence<br><br>CAS-TypeIIU	3315	6392	1	cas9_TypeII		

Figure S3 The genome map (A) and linear genome sequence (B) of *N. mangyaensis* NH1, predicted phage genes and regions positioned relative to each other across the entire genome.

A

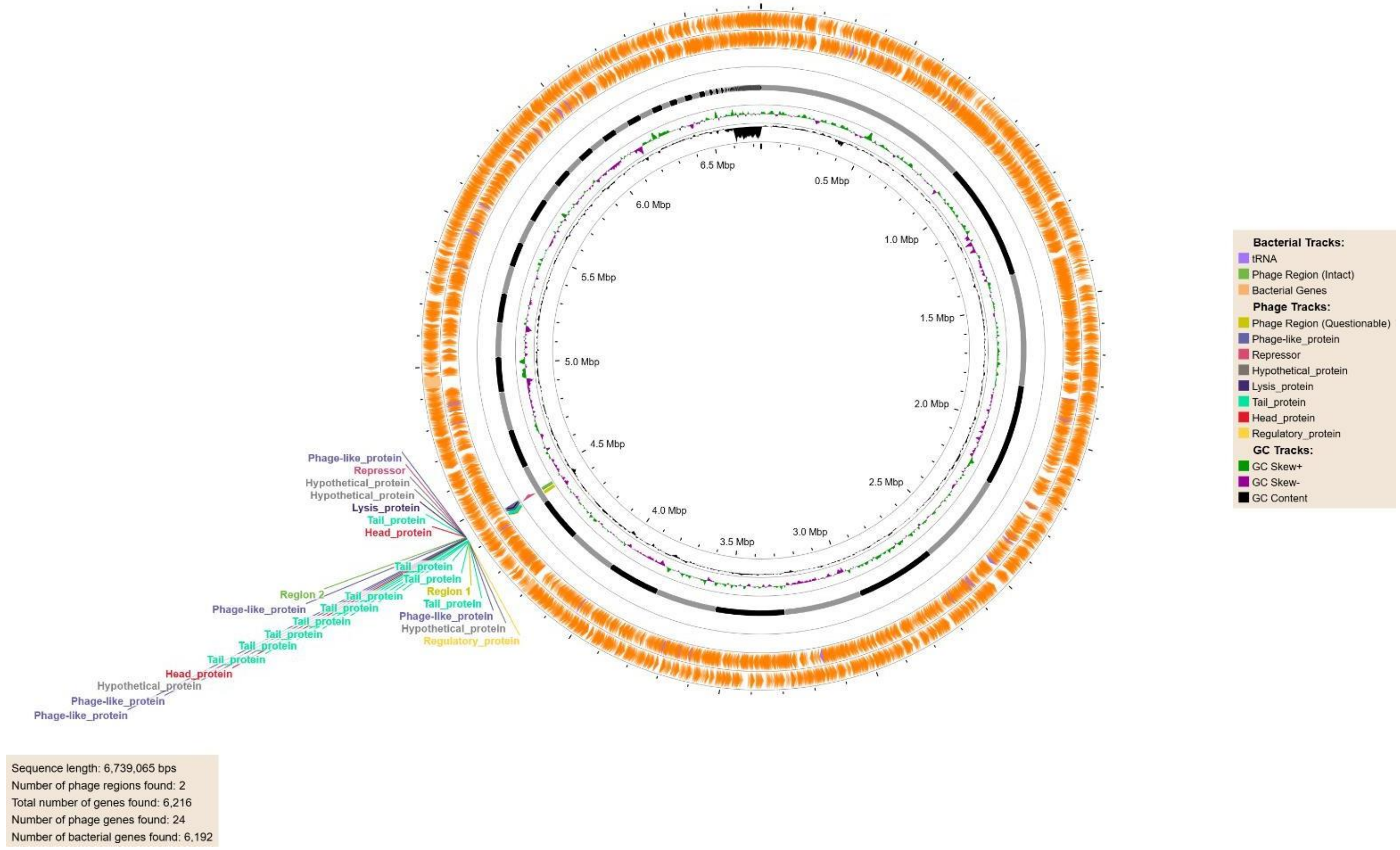

B

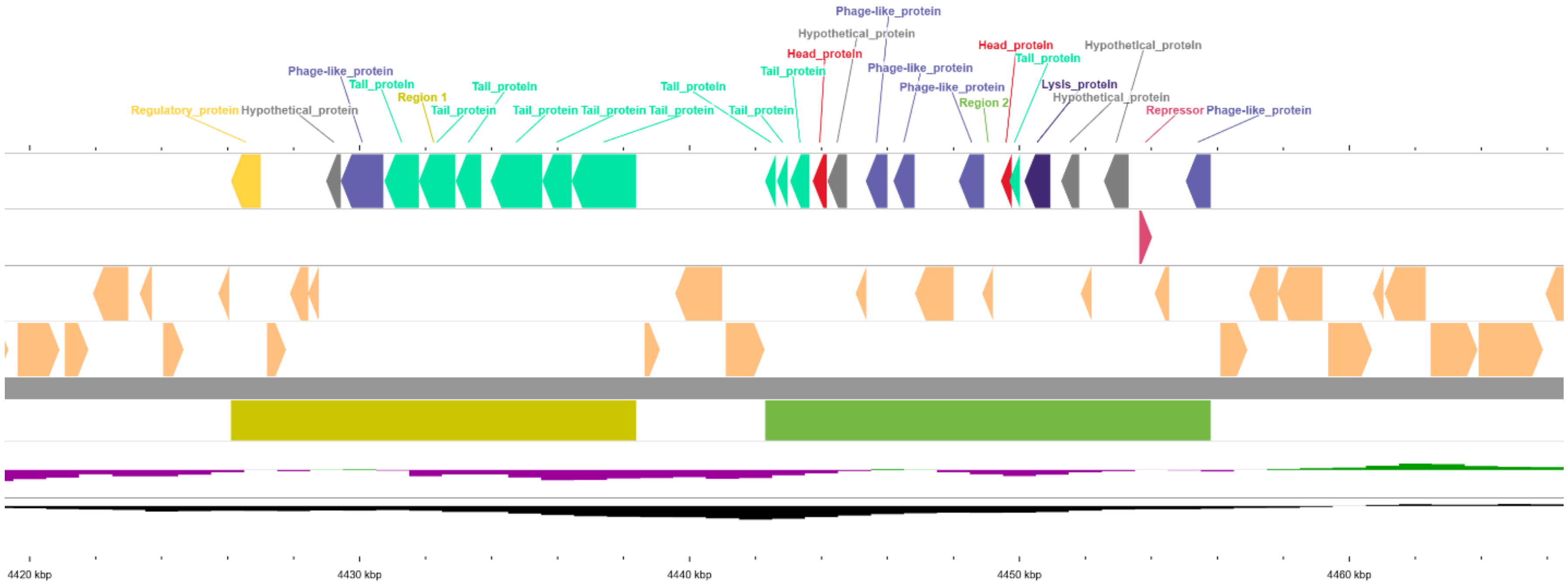

Phage-like_protein
Hypothetical_protein
Head_protein
Head_protein
Hypothetical_protein
Tail_protein
Tail_protein
Phage-like_protein
Phage-like_protein
Tail_protein
Tail_protein
Phage-like_protein
Phage-like_protein
Region 1
Region 2
Lysis_protein
Hypothetical_protein
Regulatory_protein
Hypothetical_protein
Tail_protein
Tail_protein
Tail_protein
Tail_protein
Tail_protein
Repressor
Phage-like_protein
4420 kbp
4430 kbp
4440 kbp
4450 kbp
4460 kbp

Table S5 The prediction of predict prophage sequences in the genome of *N. mangyaensis* NH1 was performed by using a web server PHASTEST

Region	Region Length	Completeness	Score	# Total Proteins	Region Position	Most Common Phage	GC %	Details
1	12.2Kb	questionable	70	12	27949-<br>40233 info_outline	PHAGE_Gordon_BrutonGaster_NC_048169(3)	64.06%	PHAGE_Gordon_BrutonGaster_NC_048169(3)<br>PHAGE_Gordon_Cucurbita_NC_031029(2)<br>PHAGE_Gordon_Bantam_NC_031074(2)<br>PHAGE_Gordon_OneUp_NC_030917(2)<br>PHAGE_Gordon_Bachita_NC_030936(2)<br>PHAGE_Coryne_Stiles_NC_048789(1)<br>PHAGE_Paraco_vB_PmaS_IMEP1_NC_026608(1)<br>PHAGE_Coryne_Lederberg_NC_048790(1)<br>PHAGE_Mycoba_Spud_NC_011270(1)<br>PHAGE_Gordon_Smoothie_NC_030696(1)<br>PHAGE_Rhodoc_REQ3_NC_016654(1)<br>PHAGE_Mycoba_Nappy_NC_023725(1)<br>PHAGE_Mycoba_Ximenita_NC_051603(1)<br>PHAGE_Bordet_vB_BbrM_PHB04_NC_047861(1)<br>PHAGE_Mycoba_Cali_NC_011271(1)<br>PHAGE_Coryne_Adelaide_NC_048791(1)<br>PHAGE_Gordon_Daredevil_NC_048021(1)<br>PHAGE_Mycoba_Keshu_NC_026603(1)<br>PHAGE_Gordon_Yvonnetastic_NC_031230(1)<br>PHAGE_Mycoba_ShedlockHolmes_NC_028846(1)<br>PHAGE_Mycoba_Gizmo_NC_021346(1)<br>PHAGE_Gordon_ClubL_NC_030901(1)<br>PHAGE_Coryne_SamW_NC_048069(1)<br>PHAGE_Mycoba_Macncheese_NC_042338(1)<br>PHAGE_Mycoba_ScottMcG_NC_011269(1)

2	13.5Kb	intact	100	20	44143-57651 info_outline	PHAGE_Gordon_Troje_NC_042102(2)	63.68%	PHAGE_Gordon_Troje_NC_042102(2)<br>PHAGE_Microc_MaMV_DC_NC_029002(2)<br>PHAGE_Gordon_Cozz_NC_030941(2)<br>PHAGE_Gordon_Getalong_NC_048083(2)<br>PHAGE_Gordon_Attis_NC_041883(1)<br>PHAGE_Strept_SF1_NC_028807(1)<br>PHAGE_Brevib_Jenst_NC_028805(1)<br>PHAGE_Gordon_SoilAssassin_NC_031251(1)<br>PHAGE_Rhodoc_Sleepyhead_NC_048782(1)<br>PHAGE_Mycoba_RhynO_NC_023609(1)<br>PHAGE_Arthro_Kuleana_NC_049473(1)<br>PHAGE_Mycoba_JHC117_NC_042310(1)<br>PHAGE_Rhodoc_Jace_NC_047974(1)<br>PHAGE_Gordon_Horus_NC_048039(1)<br>PHAGE_Mycoba_Xeno_NC_031243(1)<br>PHAGE_Mycoba_DS6A_NC_023744(1)<br>PHAGE_Gordon_Eyre_NC_031122(1)<br>PHAGE_Gordon_GMA4_NC_030939(1)<br>PHAGE_Gordon_Phistory_NC_048040(1)<br>PHAGE_Strept_SF3_NC_028952(1)<br>PHAGE_Edward_MSW_3_NC_020082(1)<br>PHAGE_Gordon_Fairfaxidum_NC_048185(1)<br>PHAGE_Rhodoc_REQ3_NC_016654(1)<br>PHAGE_Gordon_Bantam_NC_031074(1)<br>PHAGE_Gordon_GTE2_NC_015720(1)<br>PHAGE_Mycoba_Phantastic_NC_024148(1)<br>PHAGE_Gordon_Daredevil_NC_048021(1)<br>PHAGE_Mycoba_Twister_NC_041982(1)<br>PHAGE_Arthro_Andrew_NC_048098(1)<br>PHAGE_Mycoba_Gaia_NC_026590(1)<br>PHAGE_Gordon_Tanis_NC_048817(1)<br>PHAGE_Mycoba_Seagreen_NC_028813(1)<br>PHAGE_Gordon_Asapag_NC_048146(1)<br>PHAGE_Mycoba_Bipper_NC_031253(1)<br>PHAGE_Strept_Rowa_NC_047906(1)<br>PHAGE_Mycoba_Rebeuca_NC_042341(1)<br>PHAGE_Mycoba_Phrann_NC_031266(1)<br>PHAGE_Arthro_HunterDalle_NC_041941(1)<br>PHAGE_Gordon_Secretariat_NC_048876(1)<br>PHAGE_Mycoba_SkinnyPete_NC_041882(1)
